# Comparison of fluctuations in global network topology of modeled and empirical brain functional connectivity

**DOI:** 10.1371/journal.pcbi.1006497

**Published:** 2018-09-25

**Authors:** Makoto Fukushima, Olaf Sporns

**Affiliations:** 1 Center for Information and Neural Networks, National Institute of Information and Communications Technology, Suita, Osaka, Japan; 2 Graduate School of Frontier Biosciences, Osaka University, Suita, Osaka, Japan; 3 Department of Psychological and Brain Sciences, Indiana University, Bloomington, Indiana, United States of America; 4 Indiana University Network Science Institute, Bloomington, Indiana, United States of America; Ghent University, BELGIUM

## Abstract

Dynamic models of large-scale brain activity have been used for reproducing many empirical findings on human brain functional connectivity. Features that have been shown to be reproducible by comparing modeled to empirical data include functional connectivity measured over several minutes of resting-state functional magnetic resonance imaging, as well as its time-resolved fluctuations on a time scale of tens of seconds. However, comparison of modeled and empirical data has not been conducted yet for fluctuations in global network topology of functional connectivity, such as fluctuations between segregated and integrated topology or between high and low modularity topology. Since these global network-level fluctuations have been shown to be related to human cognition and behavior, there is an emerging need for clarifying their reproducibility with computational models. To address this problem, we directly compared fluctuations in global network topology of functional connectivity between modeled and empirical data, and clarified the degree to which a stationary model of spontaneous brain dynamics can reproduce the empirically observed fluctuations. Modeled fluctuations were simulated using a system of coupled phase oscillators wired according to brain structural connectivity. By performing model parameter search, we found that modeled fluctuations in global metrics quantifying network integration and modularity had more than 80% of magnitudes of those observed in the empirical data. Temporal properties of network states determined based on fluctuations in these metrics were also found to be reproducible, although their spatial patterns in functional connectivity did not perfectly matched. These results suggest that stationary models simulating resting-state activity can reproduce the magnitude of empirical fluctuations in segregation and integration, whereas additional factors, such as active mechanisms controlling non-stationary dynamics and/or greater accuracy of mapping brain structural connectivity, would be necessary for fully reproducing the spatial patterning associated with these fluctuations.

## Author summary

In human neuroscience, there is growing interest in temporal fluctuations in coactivation patterns of resting-state brain activity. To elucidate generative mechanisms of these fluctuations, theoretical studies try to reproduce their empirical properties by simulations using dynamic models of large-scale spontaneous brain activity. However, evaluations of the reproducibility have not been extended so far to the fluctuations in global network topology of coactivation patterns, recently shown to be related to human cognition and behavior. Here we examine the extent to which a stationary model typically used for simulating resting-state activity can reproduce spatial and temporal patterns of the empirically observed fluctuations in global network topology. We found that such a model successfully reproduced the magnitude of empirical fluctuations as well as their temporal dynamics, whereas their spatial patterning was not fully accounted for by the simulation. Our results suggest that stationary models can explain many empirical properties in the fluctuations in global network topology, while modeling of non-stationary dynamics and/or greater estimation accuracy of anatomical connections underlying the simulation would be required for complete replication. This finding provides new insights into how fluctuations in global network topology of coactivation patterns emerge in the human brain.

## Introduction

Neural elements in the brain are structurally connected and functionally coupled heterogeneously to form complex networks, in which neurons, neuronal populations, or brain regions can be viewed as nodes linked by edges of structural connectivity and functional connectivity [1, 2]. Structural connectivity refers to a pattern of anatomical connections between neural elements [3], defining the “wiring diagram.” On the other hand, functional connectivity refers to a pattern of statistical dependence among activities of neural elements [4], which, in human neuroimaging, has typically been assessed by the blood oxygenation level dependent (BOLD) signal measured over several minutes of resting-state functional magnetic resonance imaging (rs-fMRI) [5]. Recent advancements in measurement and analysis of rs-fMRI data allow tracking fluctuations in functional connectivity on a time scale of tens of seconds [6–9]. Fluctuations in such time-resolved functional connectivity have been found not only at the individual edge level, but also at the global network level; for example, fluctuations between segregated and integrated network topology [10] and fluctuations between high and low modularity topology [11, 12]. Fluctuations in global network topology of time-resolved functional connectivity have been associated with various types of human behavior, e.g., pupil dilation [10] and eyelid closures [13] during rest, as well as cognitive performance [10] and decoding accuracy [14] during tasks.

Along with empirical studies, a number of efforts have been made to model collective neural behavior in large-scale cortical systems [15], such as regional cortical activity during rest, and there is an increasing availability of computational tools to support these modeling practices [16, 17]. Simulating resting-state cortical activity using a set of nonlinear dynamic models wired according to structural connectivity generates synthetic BOLD time series that can be processed to yield functional connectivity, illustrating relations between anatomy and brain dynamics [18, 19]. Modeling of rs-fMRI-based functional connectivity has also been performed with other types of dynamic models of large-scale brain activity [20–23]. Furthermore, modeling efforts have recently been extended to fluctuations in time-resolved functional connectivity to reproduce salient empirical findings [9, 24–27]. Features of functional connectivity being explained by model simulations are comprehensively reviewed in [28].

While fluctuations in modeled time-resolved functional connectivity have started to be investigated, the reproducibility of fluctuations in its global network topology has not been comprehensively evaluated. Existing studies have shown that model simulations can generate fluctuations in integrated topology [29] as well as in global network efficiency [8] (a network metric closely related to modularity), but fluctuations in these global network configurations have not yet been directly compared to the empirical counterparts. Specifically, neither a detailed model parameter search using empirical data nor an examination of the degree to which empirical fluctuations can be predicted by the model has been performed. Moreover, no comparison has been conducted so far for many spatial and temporal features associated with network states determined based on fluctuations between segregated and integrated topology [10] or high and low modularity topology [12]. Examples of such features include the quantity of network metrics used for determining network states, temporal dynamics of transitions over network states, and spatial patterns of functional connectivity during each network state. In addition, it remains unclear if some or all aspects of empirical fluctuations can be accounted for by emerging properties of nonlinear stationary dynamics or require active physiological mechanisms, e.g. to trigger transitions between networks states.

To address these gaps in the literature, we compared fluctuations in global network topology of modeled and empirical functional connectivity and examined the reproducibility of empirical findings. We modeled regional resting-state cortical activity using a variant of the Kuramoto model [30, 31], a coupled phase oscillator system in which each oscillator was linked to each other based on brain structural connectivity, and generated modeled BOLD signal using the Balloon/Windkessel hemodynamic model [32]. With this stationary model of spontaneous brain activity, we evaluated fluctuations in global network topology of modeled functional connectivity by comparing their magnitude to that derived from empirical functional connectivity and searched (fitted) model parameters, such as the global coupling constant and the conduction velocity, based on this evaluation. With the model parameters selected, we compared network states of functional connectivity (segregated and integrated states [10]; high and low modularity periods [12]) between the modeled and empirical data. We first checked the reproducibility of network metrics used for determining network states, as well as the reproducibility of temporal metrics characterizing the transition dynamics of network states. We then examined whether spatial patterns of functional connectivity during each network state are reproducible or not in the modeled data. We particularly focused on examining the reproducibility of empirical findings regarding spatial connectivity patterns in previous studies [12, 33], where we reported characteristic between-state changes in functional connectivity within/between task-positive and task-negative networks and in the similarity between structural connectivity and functional connectivity. Through these comparisons, we demonstrated which empirical features of fluctuations in segregation and integration can be reproduced by a stationary dynamic model typically used for simulating resting-state brain activity.

## Materials and methods

### Data set

Imaging data in this study are from the data sample labeled *100 Unrelated Subjects* in ConnectomeDB (https://db.humanconnectome.org), the database managed by the Washington University-University of Minnesota (WU-Minn) consortium of the Human Connectome Project (HCP; http://www.humanconnectome.org). Participants were recruited by the WU-Minn HCP consortium and provided written informed consent prior to experiments [34]. All experimental procedures were approved by the Institutional Review Board (IRB) at Washington University (IRB number 201204036; “Mapping the Human Connectome: Structure, Function, and Heritability”) and no further IRB approval is required for our data analysis. From this data sample, we first discarded 15 subjects because of their large head movements during acquisitions of rs-fMRI data. Subjects were excluded when maximum translation exceeded 3 mm, maximum rotation exceeded 3°, or mean framewise displacement (FD; the *l*2 norm version) exceeded 0.2 mm [35] in at least one run of rs-fMRI acquisition. We additionally excluded one subject aged ≥ 36 years to obtain a sample of young adults aged ≥ 22 years and < 36 years. The final number of subjects in this sample was 84 (male, 40; female, 44).

All MRI data in this data sample were acquired with a 32-channel head coil on a modified 3T Siemens Skyra. Scanning parameters of acquired T1-weighted structural images were: repetition time (TR) = 2,400 ms, echo time (TE) = 2.14 ms, flip angle = 8°, field of view (FOV) = 224 × 224 mm^2^, 320 slices, and voxel size = 0.7 mm isotropic. The rs-fMRI data in this sample were acquired with the following parameters: TR = 720 ms, TE = 33.1 ms, flip angle = 52°, FOV = 208 × 180 mm^2^, 72 slices, and voxel size = 2 mm isotropic. For all 84 subjects, four runs of rs-fMRI data were collected with an eyes open condition and the duration per run was around 14 min (1, 200 time points). Scanning parameters of diffusion-weighted images (DWI) were: TR = 5,520 ms, TE = 89.5 ms, flip angle = 78°, FOV = 210 × 180 mm^2^, 111 slices, and voxel size = 1.25 mm isotropic (three shells, two repeats, and 36 *b*0 scans). The number of gradient directions of the acquired DWI data was 270 and the *b*-value was 1, 000, 2, 000, and 3, 000 s/mm^2^ for each of the three shells.

### Data preprocessing

The data sample downloaded from the ConnectomeDB has already been preprocessed with the minimal preprocessing pipeline of the HCP [36]. Preprocessing steps for rs-fMRI data included in this pipeline were: correction of gradient distortion, motion correction, removal of bias fields, correction of spatial distortion, transformation to Montreal Neurological Institute (MNI) space, and normalization of the image intensity. Preprocessing steps for DWI data included normalization of the intensity and corrections of susceptibility distortion, eddy current distortion, motion-related artifact, and gradient nonlinearly.

To further improve data quality, we additionally preprocessed the rs-fMRI data by taking the following steps: (a) removal of the first 10 s of volumes, (b) removal of outlier volumes and interpolation (the percentage of interpolated volumes was 3.6 ± 0.1% [mean ± SD across all subjects and runs]), (c) regressing out the Friston-24 motion time series [37] and the global, white matter, and cerebrospinal fluid mean signals, and (d) detrending and band-pass filtering (cutoff frequency: (66 TRs)^−1^ = 0.021 Hz [low], 0.1 Hz [high]). To exclude spurious fluctuations, we specified the low-cut frequency of the band-pass filtering to the reciprocal of the width of the sliding window for time-resolved functional connectivity [38, 39]. The outlier removal and interpolation in step (b) were performed using *3dDespike* in the AFNI package [40] as in [41]. The removal of outlier volumes is similar to motion scrubbing and censoring [42, 43], but we replaced outliers with interpolated volumes instead of discarding affected time points, in order to keep the original number of time points within a sliding window over the whole time course. We chose to include global signal regression in step (c) to remove global artifacts that are attributable to motion and/or respiration [44].

White matter fiber tracts were reconstructed from the DWI data using generalized *q*-sampling imaging [45] and deterministic streamline tractography. The use of the generalized *q*-sampling imaging method allows for the reconstruction of complex fiber configurations. Details of the procedure of tractography are presented in [46–48].

### Cortical parcellation

Connectivity analyses were performed in a region-wise manner within the cortex. Nodes of connectivity networks in this study were assigned to each of the 114 distinct cortical parcels, made by a subdivision of the Desikan-Killiany atlas [49] (see S1 Fig). These subdivided parcels were obtained from the atlas files *myatlas_60_lh.gcs* and *myatlas_60_rh.gcs* in the Connectome Mapper package (https://github.com/LTS5/cmp). In addition, we assigned every node (parcel) to one of the seven intrinsic connectivity networks defined in [50] by evaluating the area of overlap of cortical surface. These seven networks are named as follows: the control network (CON), the default mode network (DMN), the limbic system (LIM), the dorsal attention network (DAN), the saliency/ventral attention network (VAN), the somatomotor network (SMN), and the visual network (VIS).

### Structural connectivity

**Fig 1:**
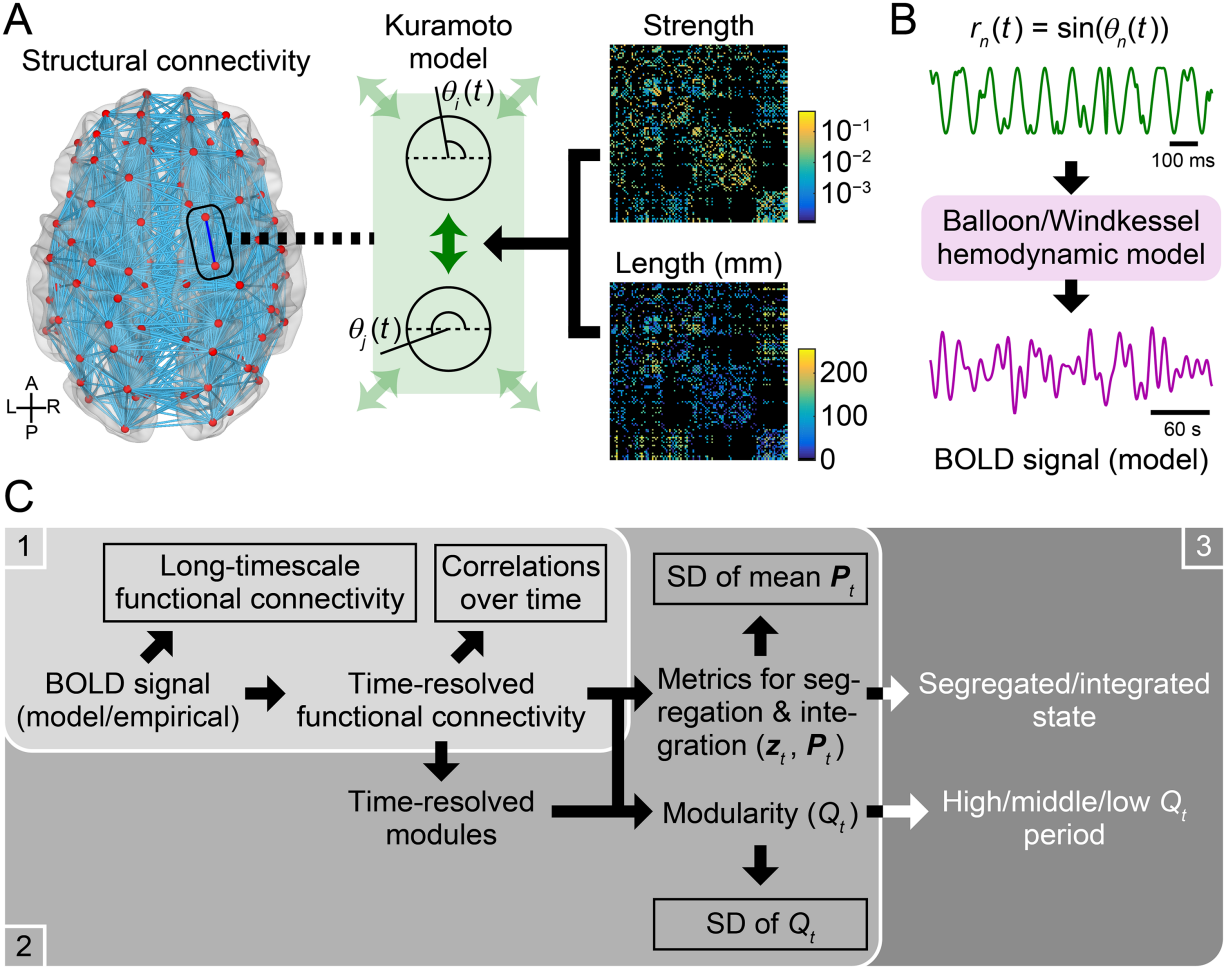
Workflow diagrams for model simulation and parameter search. (A) A schematic of simulating resting-state cortical activity using a system of coupled phase oscillators, known as the Kuramoto model. Each oscillator was assigned to a cortical node and was coupled based on connection strengths and fiber lengths of brain structural connectivity. (B) A schematic of converting modeled cortical activity to modeled BOLD signal. (C) Workflow diagram of model parameter search. 1. For all 28 × 16 parameter sets, long-timescale functional connectivity and the correlation distribution of time-resolved functional connectivity over time (framed by rectangles in the panel 1) were computed from the modeled BOLD signal and were compared to their empirical counterparts (10 simulation samples per each parameter set). 2. For the parameter sets with better matches between the modeled and empirical data in the first stage, community detection was performed on time-resolved functional connectivity to obtain its modules (100 simulation samples per each parameter set). Then, time series of mean participation coefficient ***P***_*t*_ and modularity *Q*_*t*_ were computed and their SDs (framed by rectangles in the panel 2) were compared between the modeled and empirical data. 3. For the parameter set yielding the best match between the modeled and empirical data in the second stage, changes in functional connectivity across networks states (segregated and integrated states or high, middle, and low modularity periods) were compared between the modeled and empirical data.

Structural connectivity strength between a pair of cortical regions was measured using fiber density, defined as the streamline count between the two regions divided by the geometric mean of the surface areas of these regions. We used fiber density as a strength metric of structural connectivity in order to compensate for an effect of the size of regions on streamline counts [51]. From structural connectivity in individual participants, group-level structural connectivity was derived using a consensus approach that preserves the fiber length distributions of individual-level structural connectivity within and between hemispheres, respectively [52]. For the edges selected by this consensus approach, connectivity strength and fiber length were averaged across subjects to construct group-level matrices (see Fig 1A, right), where averaging at such an edge was performed across subjects whose corresponding connectivity strength at this edge was non-zero.

### Long-timescale functional connectivity

As a metric of functional connectivity, we used the Pearson correlation coefficient between regional BOLD time courses. The correlation coefficient was Fisher *z*-transformed except when it was shown in connectivity matrices in figures. We refer to the functional connectivity measured over the entire rs-fMRI run as long-timescale functional connectivity.

### Time-resolved functional connectivity

With the metric of functional connectivity defined above, time-resolved functional connectivity was estimated using a tapered sliding window approach [7, 53]. The shape and the size of tapered time window were specified in a similar way as in [41]. Specifically, a tapered time window was constructed by convolving a rectangle of width = 47.52 s (66 TRs) with a Gaussian kernel of *σ* = 6.48 s (9 TRs). The tapered window was moved toward the end of the BOLD time series in steps of 2.16 s (3 TRs), which resulted in a total of 369 tapered windows.

### Community detection and modularity

Communities or modules in networks of time-resolved functional connectivity were detected through modularity maximization [54] using the Louvain algorithm [55]. To cope with negative functional connectivity, we employed a modularity quality function *Q* generalized for networks containing both positive and negative edge weights [56] as follows:

$$\begin{array}{c} Q=\frac{1}{\nu^{+}}\sum_{i,j}(w_{i,j}^{+}-e_{i,j}^{+})\delta_{M_{i},M_{j}}-\frac{1}{\nu^{+}+\nu^{-}}\sum_{i,j}(w_{i,j}^{-}-e_{i,j}^{-})\delta_{M_{i},M_{j}}, \end{array}$$ (1)

where $w_{i,j}^{+}=w_{i,j}$ and $w_{i,j}^{-}=0$ if the edge weight *w*_*i*,*j*_ between nodes *i* and *j* is positive, and $w_{i,j}^{+}=0$ and $w_{i,j}^{-}=-w_{i,j}$ otherwise. *δ* in this equation is the Kronecker delta, where $\delta_{M_{i},M_{j}}=1$ if nodes *i* and *j* are within the same module and $\delta_{M_{i},M_{j}}=0$ otherwise. The term $e_{i,j}^{\pm }=s_{i}^{\pm }s_{j}^{\pm }/\nu^{\pm }$, where $s_{i}^{\pm }=\sum_{j}w_{i,j}^{\pm }$ and $\nu^{\pm }=\sum_{i,j}w_{i,j}^{\pm }$, denotes the expected density of positive or negative weights in a network given a random null model. The first term in Eq (1) corresponds to a standard form of the quality function in which negative edge weights are not taken into account. Adding the second term allows to identify partitions in which negative edges are located between modules [56]. As was argued in [56], we decided to add this term when identifying partitions in functional brain networks since negatively correlated pairs of nodes are indicative that these nodes reside in different communities.

Maximization of the modularity quality function *Q* was performed using the Matlab function *community_louvain.m* in the Brain Connectivity Toolbox (BCT; http://www.brain-connectivity-toolbox.net) with the default setting of the resolution parameter *γ* = 1. Modularity maximization was applied to the adjacency matrix of functional connectivity 100 times with random initial conditions for each time window of time-resolved functional connectivity. The maximum of the quality function across trials at time window *t* was regarded as the resulting modularity score *Q*_*t*_ and its accompanying network partition as its community assignment.

### Network metrics for segregation and integration

Based on detected communities in networks, within-module degree *z*-score and participation coefficient [57] were computed for each time window of time-resolved functional connectivity. Consistent with previous work, these two network metrics were used for estimating segregated and integrated states of functional connectivity [10].

In functional brain networks, the within-module degree *z*-score measures the extent to which a node is functionally coupled with the other nodes within the same module, relative to the weighted degrees of the other nodes within this module. The within-module degree *z*-score of node *i* at time *t* is computed as

$$\begin{array}{c} z_{i,t}=\frac{\kappa_{i,M_{i,t},t}-{\bar{\kappa }}_{M_{i,t},t}}{\sigma_{M_{i,t},t}}, \end{array}$$ (2)

where $\kappa_{i,M_{i,t},t}$ denotes the weighted degree of node *i* at time *t* within its module *M*_*i*,*t*_, and ${\bar{\kappa }}_{M_{i,t},t}$ and $\sigma_{M_{i,t},t}$ are the mean and SD of the nodal weighted degrees at time *t* within module *M*_*i*,*t*_. This network metric was computed using the function *module_degree_zscore.m* in the BCT toolbox.

The participation coefficient for functional connectivity quantifies the extent to which a node is functionally coupled with other nodes across diverse modules. The participation coefficient of node *i* at time *t* is given by

$$\begin{array}{c} P_{i,t}=1-\sum_{m=1}^{N_{t}}{\left(\frac{\kappa_{i,m,t}^{+}}{\kappa_{i,t}^{+}}\right)}^{2}, \end{array}$$ (3)

where *N*_*t*_ is the number of detected modules at time *t*, $\kappa_{i,m,t}^{+}$ is the weighted degree of the positive edge weights of node *i* at time *t* within module *m*, and $\kappa_{i,t}^{+}$ is the weighted degree of the positive edge weights of node *i* at time *t* across all modules. This metric was computed using the BCT function *participation_coef_sign.m*.

### Estimation of network states

#### Segregated and integrated states

Segregated and integrated network states of functional connectivity were estimated from a joint histogram of the within-module degree *z*-score ***z***_*t*_ and the participation coefficient ***P***_*t*_ over all nodes in the same manner as in [10, 33]. The joint histogram of these two network metrics at time *t* was constructed by summing the instances of each value of ***z***_*t*_ and ***P***_*t*_ in 100 equally defined bins along each axis (range: −5 < ***z***_*t*_ < 5 and 0 < ***P***_*t*_ < 1). Estimation of the segregated and integrated states was performed by classifying the joint histogram of each time window using *k*-means clustering with the number of clusters set to two. Applying *k*-means clustering was repeated 500 times with random initial conditions in each run in each individual. After the clustering, each time window was assigned to one of the two estimated clusters, where the cluster having greater participation coefficients on average was regarded as the cluster of the integrated state.

#### High, middle, and low modularity periods

Network states of functional connectivity were also defined using modularity *Q*_*t*_ in the present study. In keeping with a previous study, each time window of time-resolved functional connectivity was classified into either of three categories based on the intensity of modularity *Q*_*t*_ [12] (high, middle, and low modularity periods). For the sake of computational efficiency, the high and low modularity periods of functional connectivity were simply determined as periods of *Q*_*t*_ greater than its upper tercile and less than its lower tercile, respectively, over time in each run in each individual.

### Modeling regional resting-state activity

Regional resting-state cortical activity was simulated using a system of coupled phase oscillators, called the Kuramoto model [30, 31, 58, 59]. We selected the Kuramoto model to simulate resting activity because it is simple enough to be tractable, while it can simulate synchronization behavior in neural dynamics by taking into account delays between oscillators [60] and, more importantly, can reproduce empirical findings about functional connectivity in the resting brain by linking oscillators based on structural connectivity [20, 23, 61–63]. While several recent studies directly model slow BOLD fluctuations using the Kuramoto model [9, 64, 65], we adopted the approach taken by [20, 23, 62, 63], where the Kuramoto model is used for simulating fast oscillatory activity of neural populations in the gamma frequency band (30–90 Hz) [66] and then the simulated neural activity is converted to modeled BOLD time series using a hemodynamic model. Experimental studies have shown that fluctuations in the gamma-band power of neural activity are closely related to spontaneous BOLD signal [67–70].

The periodical dynamic behavior of node *i* in the network of coupled oscillators is described using its phase *θ*_*i*_(*t*), and it obeys the following differential equation:

$$\begin{array}{c} \frac{d\theta_{i}}{dt}=2\pi f+k\sum_{j=1}^{N}C_{i,j}\sin\;(\theta_{j}\left(t-\tau_{i,j}\right)-\theta_{i}\left(t\right)), \end{array}$$ (4)

where *f* denotes the natural frequency, which was set to 60 Hz for all nodes [23, 63]. The second term in this equation represents the influences from the other nodes that are structurally connected to node *i*. In this term, *N* denotes the number of nodes, *k* is the global coupling constant that controls the overall strength of the couplings, *C*_*i*,*j*_ is the strength of group-level structural connectivity between nodes *i* and *j*, where it was normalized so that the average of all non-zero edge weights equals one, and *τ*_*i*,*j*_ is the time delay of interactions between nodes *i* and *j*. The time delay was assumed to be proportional to the fiber length *L* between nodes *i* and *j* such that *τ*_*i*,*j*_ = *L*_*i*,*j*_/*v*, where *v* represents the conduction velocity in myelinated fibers [20, 23, 61–63]. The way to specify the model parameters *k* and *v* are described later in **Model parameter search** in **Materials and methods**. A schematic of model simulation is shown in Fig 1A.

In Eq (4), no noise was added to the system as in [61, 71]. Qualitatively similar results were obtained even when the system noise was introduced to the model simulation. The main results of this study with noise added are presented in **Supporting information** (S6 and S7 Figs). When the simulation was performed with noise, white Gaussian noise with *σ* = 1.25 rad/s was added to the system as in [23, 63].

The differential equation in Eq (4) was numerically solved using the deterministic Heun method for the simulation without noise and the stochastic Heun method for the simulation with noise. The step size of the numerical integration was set to 0.2 ms. The initial value of the phase was randomly drawn from the uniform distribution of the range [0, 2*π*]. The initial history of the phase, which must be specified due to the presence of delays, was generated by running simulations for a short duration without interactions [20, 61]. To remove transient dynamics, the initial 20 s of data were discarded from the simulated phase time series.

The global level of synchrony of the oscillator system can be evaluated using the order parameter *R*(*t*):

$$\begin{array}{c} R\left(t\right)e^{i\Phi (t)}=\frac{1}{N}\sum_{n=1}^{N}e^{i\theta_{n}\left(t\right)}, \end{array}$$ (5)

where *R*(*t*) quantifies the phase uniformity, which varies between 0 (fully incoherent) and 1 (fully synchronized), and Φ(*t*) describes the phase of the global ensemble of the oscillators. We characterized the global dynamics of the system using the mean and the SD of the order parameter *R*(*t*), which measure respectively the level of global synchrony and the level of global metastability of the whole system [72, 73].

After transforming the phase time courses *θ*_*n*_(*t*) into the simulated regional activities *r*_*n*_(*t*) as *r*_*n*_(*t*) = sin(*θ*_*n*_(*t*)) (*n* = 1, …, *N*) [20] and downsampling them to the sampling frequency of 1 kHz, these regional activity data were converted to the BOLD time courses using the Balloon/Windkessel hemodynamic model [32] with the parameter setting used in [74] (Fig 1B). The obtained modeled BOLD signal was preprocessed using the same band-pass filter that was applied to the empirical rs-fMRI data. Additionally, the modeled data were downsampled to make their TR identical to the empirical data, and the global signal was regressed out as was performed for the empirical data. The number of time points in a single simulation sample of the modeled data was set to be identical to that of a single run of the preprocessed empirical rs-fMRI data (approximately 14 min). For each simulation sample of the modeled data, computation of long-timescale and time-resolved functional connectivity, community detection, calculation of the network metrics *Q*_*t*_, ***z***_*t*_ and ***P***_*t*_, and the estimation of the network states were conducted in the same manner as for the empirical data.

In addition to relating to the empirical data, the magnitude of fluctuations in global network topology of modeled functional connectivity was compared to that obtained from the model simulations with surrogate structural connectivity. The surrogate connectivity data were constructed by randomly rewiring edges with preserving the degree and the weighted degree of each node. The rewiring of edges was performed using the BCT function *null_model_und_sign.m* with the default setting. We kept generating surrogates until we obtained 100 random samples whose correlation coefficient between weighted-degree sequences of actual and surrogate connection matrices was greater than 0.95. We also controlled the level of global synchrony of simulated activity by performing model parameter search in each surrogate sample. Details of this procedure are presented in the last paragraph of the next section **Model parameter search**.

### Model parameter search

The global coupling constant *k* and the conduction velocity *v* were searched so that long-timescale functional connectivity, time-resolved functional connectivity, and fluctuations in its global network topology of the modeled data become close to those obtained from the empirical data. As in [20, 61, 62], the conduction velocity *v* was searched through changing the mean time delay $\bar{\tau }=\bar{L}/v$, where $\bar{L}$ denotes the mean fiber length ($\bar{L}=84.5$ mm in our data). The search for the model parameters *k* and $\bar{\tau }$ was performed in two stages in the following manner.

#### First stage

In this stage, we ran the model simulation 10 times per each of a large number of parameter sets and evaluated the similarity of the modeled and empirical data both in terms of long-timescale and time-resolved functional connectivity (Fig 1C, panel 1). The global coupling constant *k* was searched over the range of 2.5–70 in steps of 2.5, and the mean time delay $\bar{\tau }$ was changed from 2 to 17 ms in steps of 1 ms (this corresponds to changing the conduction velocity *v* in the range of 42.3–5.0 m/s). The total number of parameter sets to be explored was 28 × 16 = 448.

The similarity of long-timescale functional connectivity was quantified using the correlation coefficient between modeled and empirical long-timescale functional connectivity weights, and was evaluated over all pairs of nodes [62], as well as over pairs of nodes with direct structural connections [20]. These similarity scores were computed from modeled long-timescale functional connectivity averaged over all simulation samples and empirical long-timescale functional connectivity averaged over all rs-fMRI runs and subjects.

Comparison of modeled and empirical time-resolved functional connectivity was performed using the distribution of the correlation coefficients of time-resolved connectivity itself over time, which has been referred to as functional connectivity dynamics (FCD; [24]). The correlation distributions were constructed by stacking the correlation coefficients of time-resolved connectivity within each sample or run one by one. The correlations among time-resolved connectivity within the width of time window were discarded from the resulting distributions. The (dis)similarity of the correlation distributions between the modeled and empirical data was measured using the Kolmogorov-Smirnov (KS) distance.

We extracted a small number of parameter sets yielding better scores of these evaluation measures and then further examined their reproducibility of empirical findings. Parameter sets were extracted for further analyses if they exhibited the correlation coefficient of modeled and empirical long-timescale functional connectivity with direct structural connections greater than 0.33 and the KS distance of the correlation distributions of modeled and empirical time-resolved functional connectivity over time less than 0.33.

#### Second stage

In the second stage, we newly generated 100 samples of simulation data for each of the parameter sets extracted in the first stage. With these large number of simulation samples, we examined the similarity of fluctuations in global network topology of modeled and empirical functional connectivity (Fig 1C, panel 2).

We compared modeled and empirical fluctuations in time series of mean participation coefficient ***P***_*t*_ over nodes and modularity *Q*_*t*_. Mean ***P***_*t*_ has been shown to be closely correlated with transitions between the segregated and the integrated network states [10]. *Q*_*t*_ was directly used for determining the high, middle, and low modularity periods as explained above. The similarity of modeled and empirical fluctuations in these global network metrics that are associated with network states was quantified using the ratio of each metric’s SD over time within each sample of the modeled data to that averaged across runs and subjects in the empirical data.

We selected the parameter set yielding the highest similarity of modeled and empirical fluctuations in network integration (mean ***P***_*t*_) and modularity (*Q*_*t*_). With the parameter set selected, we compared network states between the modeled and empirical data as explained below in the following section.

When comparing the magnitude of fluctuations in global network topology between the actual and surrogate modeled data, we controlled the level of global synchrony by fitting the mean of the order parameter through model parameter search. For the sake of computational efficiency, we fixed the mean time delay $\bar{\tau }$ to the same value as in the actual data and varied the global coupling constant *k* for each surrogate connectivity data. The parameter *k* was changed over the range of 2.5–70 in steps of 2.5 and picked up a value from which the mean of the order parameter was the closest to that of the actual data.

### Comparison of network states

After the model parameter search, we compared network states of functional connectivity (segregated and integrated states or high, middle, and low modularity periods) between the modeled and empirical data (Fig 1C, panel 3). In particular, we compared network metrics that were used for determining network states (i.e., *Q*_*t*_, ***z***_*t*_, and ***P***_*t*_), temporal metrics that characterize the dynamics of network states (transition probability and mean dwell time), and spatial patterns of functional connectivity during each network state.

When comparing spatial patterns of modeled and empirical functional connectivity, we investigated changes in functional connectivity across network states through the centroids of time-resolved functional connectivity during each network state. Edge weights of a centroid were computed as the median of time-resolved functional connectivity over time during the corresponding network state within each sample or run [41]. Centroids of the empirical data were averaged over all four rs-fMRI runs within each individual. With these centroids, we examined whether empirical findings about the following two characteristic changes in functional connectivity across network states, reported in [12, 33], are reproducible or not in the modeled connectivity data.

- Changes in functional connectivity within/between task-positive and task-negative networks [12]. Functional connectivity weights in each centroid were averaged within each pair of the seven intrinsic networks in [50] and differences in them between network states (segregated minus integrated, high modularity minus low modularity) were examined in terms of task-positive and task-negative systems.
- Changes in the similarity between structural connectivity and functional connectivity [33]. The similarity was measured using the correlation coefficient between non-zero edge weights of group-level structural connectivity and the centroid edge weights of functional connectivity for each network state.

## Results

### Model parameter search

#### First stage

**Fig 2:**
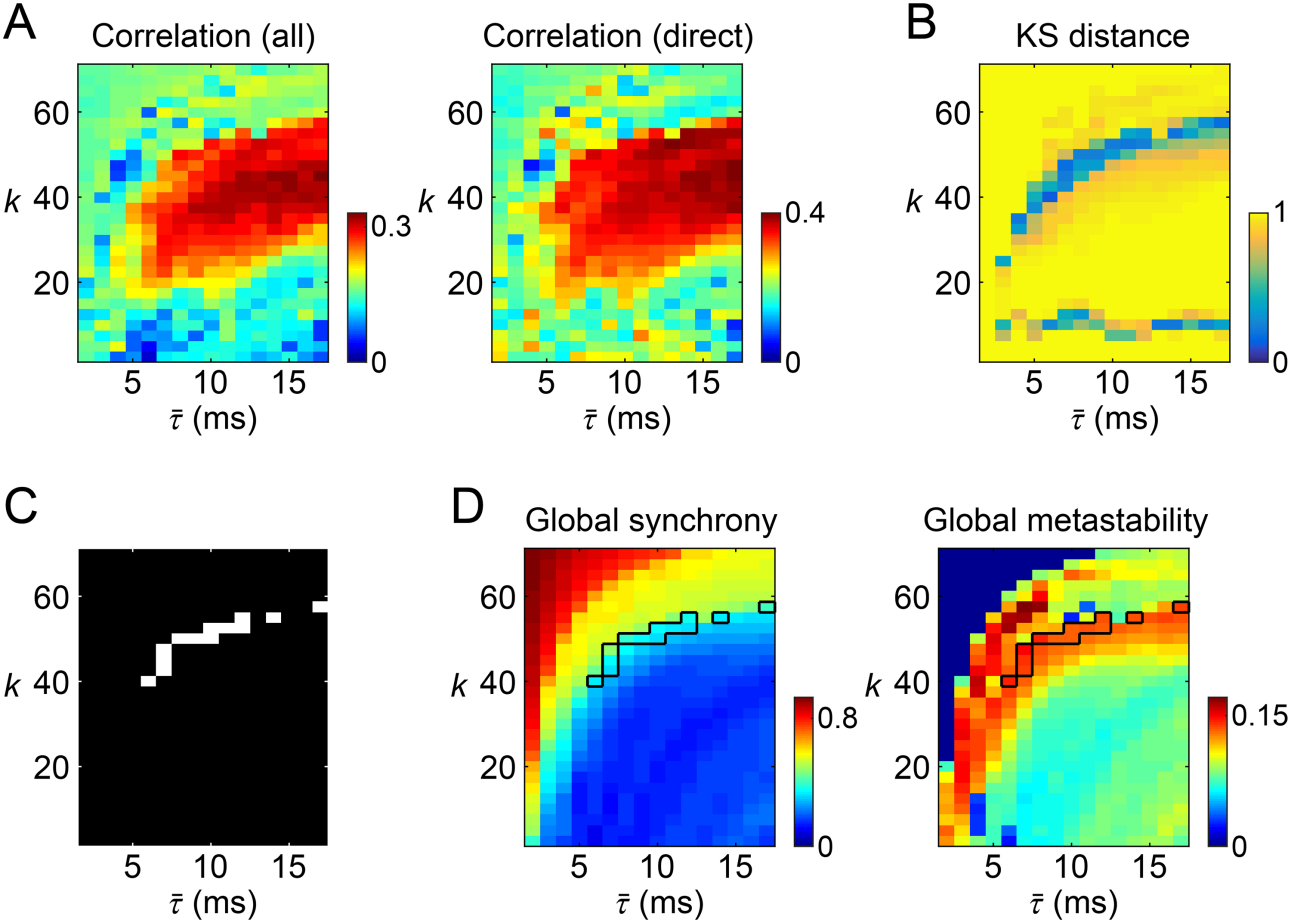
Model parameter search in the first stage. (A) Heat maps of the correlation coefficients of modeled and empirical long-timescale functional connectivity. Left: correlations evaluated over all pairs of nodes. Right: correlations evaluated over pairs of nodes with direct structural connections. (B) A heat map of the KS distance between the correlation distributions of modeled and empirical time-resolved functional connectivity over time. (C) The parameter sets extracted in the first stage based on the similarity of modeled and empirical long-timescale/time-resolved functional connectivity (see text in for selection criteria). (D) Left: a heat map of the global synchrony (the mean of the order parameter). Right: a heat map of the global metastability (the SD of the order parameter). The averages over the 10 simulation samples are presented. The parameter sets extracted in the first stage were outlined in these heat maps.

The correlation coefficients of modeled and empirical long-timescale functional connectivity are shown in Fig 2A for all parameter sets explored. The maximum correlations over parameter sets were 0.331 for all pairs of nodes and 0.400 for pairs of nodes with direct structural connections, similar in magnitude to those observed in [62] and [20], respectively. Similar heat map patterns were observed for both settings of the correlation coefficient. A necessary condition for obtaining larger correlations was setting the global coupling constant *k* near the middle of the range (22.5–57.5) of the *k*-axis and setting the mean time delay $\bar{\tau }$ to be greater than 6 ms.

Fig 2B shows the KS distance between the correlation distributions of modeled and empirical time-resolved functional connectivity over time. In a large portion of the parameter space, the KS distance was close to one, which means that the correlation distributions were totally different between the modeled and empirical data. Shorter KS distances, or more similar correlation distributions, were obtained only from a limited number of parameter sets. Such parameter sets traced a curve and a line in the heat map in Fig 2B. Among them, only the curve-shaped cluster of the parameter sets overlapped with the area of significant similarity of long-timescale functional connectivity shown in Fig 2A. Parameter sets associated with the correlations in Fig 2A (right) greater than 0.33 and the KS distances in Fig 2B less than 0.33 are shown in Fig 2C and were extracted from the large parameter space to further examine their ability to reproduce empirical findings.

Before proceeding to the second stage, we checked the global synchrony and the global metastability of resting-state cortical activity simulated with these extracted parameter sets (Fig 2D). Within simulation samples of these parameter sets, the global synchrony was 0.353 ± 0.046 and the global metastability was 0.138 ± 0.005 (mean ± SD). The observed moderate levels of the global synchrony, compared to that with other parameter sets (see the heat map in Fig 2D left), indicates that oscillators in the system were either not fully coupled with each other or randomly fluctuating in the model simulations with the extracted parameter sets. Instead, the relatively large global metastability (see Fig 2D right) suggests that synchronization and desynchronization coexist over the entire period of simulated activity generated from the extracted parameter sets. Such metastable network dynamics have also been observed with the parameter sets specified in previous studies for reproducing empirical long-timescale functional connectivity [20, 62].

#### Second stage

**Fig 3:**
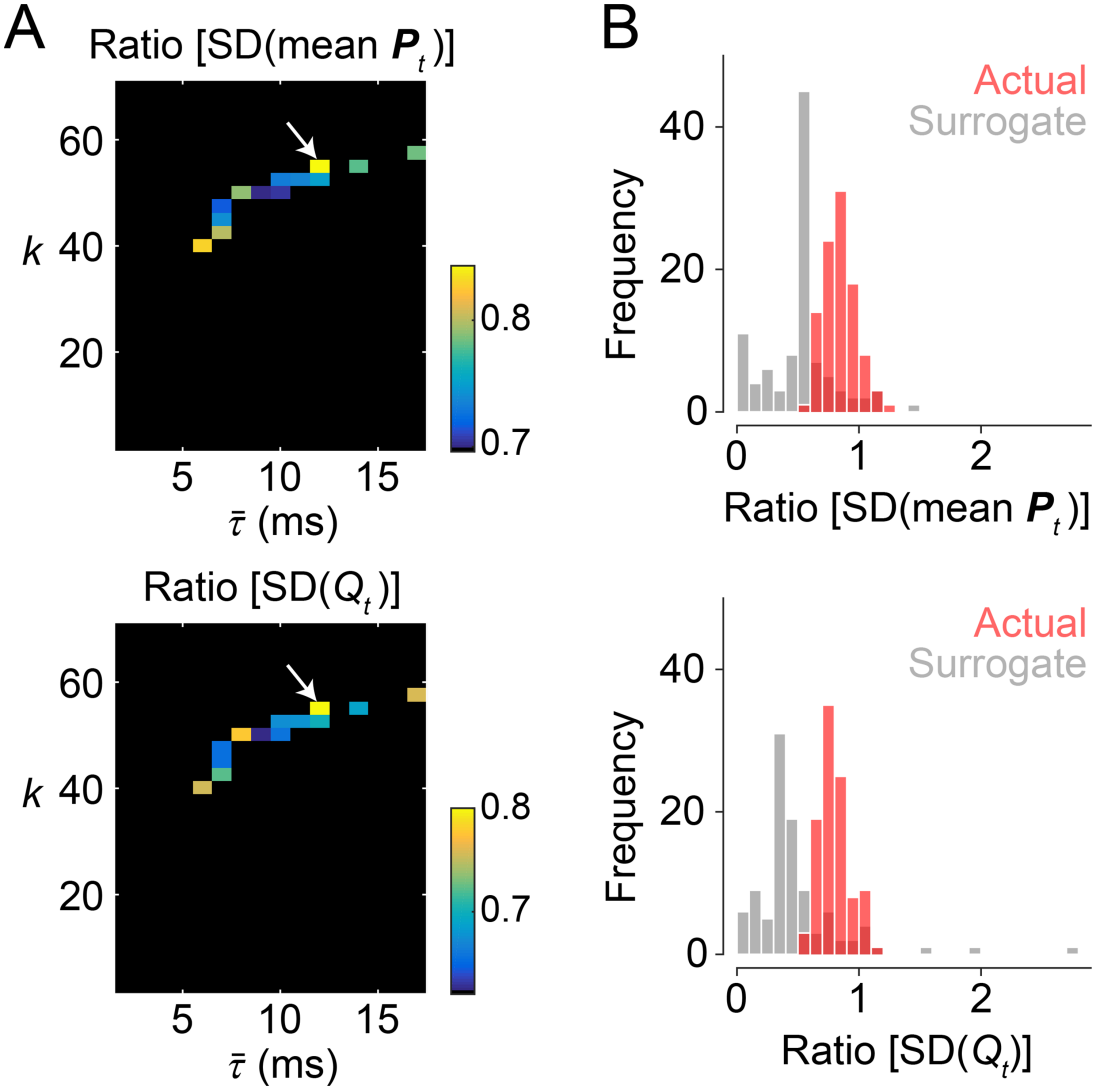
Model parameter search in the second stage. (A) The ratio of the SDs of mean participation coefficient (top) and modularity (bottom) of modeled time-resolved functional connectivity to those of the empirical ones. These ratios were evaluated at the parameter sets extracted in the first stage and their averages over the 100 simulation samples are presented in the heat maps. From the parameter set $\left(k,\bar{\tau }\right)=\left(55,12\right)$, pointed by a white arrow, we obtained the SDs of mean participation coefficient and modularity that were on average closest to those observed in the empirical data. This parameter set was selected for further comparison of the modeled and empirical data. (B) Distributions of the ratio of modeled to empirical fluctuations in mean participation coefficient (top) and modularity (bottom). The distributions obtained from simulations with the actual structural connectivity data are shown in light red and the distributions from the surrogate data are shown in light gray.

The ratio of fluctuations in global network topology of modeled functional connectivity to those of the empirical data is presented in Fig 3A. For both time series of mean participation coefficient and modularity, fluctuations measured with their SDs in the modeled data were on average closest to those observed in the empirical data when *k* = 55 and $\bar{\tau }=12$ ms. This parameter set is marked by a white arrow in the heat maps in Fig 3A. The SDs of both mean participation coefficient and modularity with this parameter set were on average greater than 80% of the magnitudes of the SDs of the empirical counterparts. In the following section, the modeled data simulated with $\left(k,\bar{\tau }\right)=\left(55,12\right)$ were used for comparison of changes in modeled and empirical functional connectivity across network states. The selected values of *k* and $\bar{\tau }$ were in a physiologically plausible range. The global coupling constant *k* is not too strong to cause the system to be unrealistically fully synchronized, as seen in Fig 2D. The mean time delay $\bar{\tau }$ corresponds to a conduction velocity 7.0 m/s, which places it within a realistic range of the conduction velocity observed for the adult primate brain (around 5–20 m/s; according to [75]).

The correlation coefficient of group-level empirical long-timescale functional connectivity to group-level structural connectivity was 0.395, and which was indeed greater than the correlation to modeled long-timescale functional connectivity averaged over 100 samples, 0.341, with the selected parameter set $\left(k,\bar{\tau }\right)=\left(55,12\right)$ (correlations evaluated over structurally connected edges). However, the model with this parameter setting added some merits above anatomy by predicting the dynamics of functional connectivity more accurately than the cases when the parameter set was specified to yield the prediction accuracy of the long-timescale functional connectivity being above that yielded by structural connectivity. The prediction accuracy of the long-timescale functional connectivity was slightly better than that obtained from structural connectivity when $\left(k,\bar{\tau }\right)=\left(52.5,13\right)$ and $\left(k,\bar{\tau }\right)=\left(45,17\right)$ (correlation coefficient of modeled and empirical long-timescale functional connectivity: 0.400 and 0.396, respectively) (Fig 2A right). With these two parameter sets, KS distances between the correlation distributions of modeled and empirical time-resolved functional connectivity over time (Fig 2B) were not as good as that obtained from the parameter set selected in the second stage. Specifying the parameter set as $\left(k,\bar{\tau }\right)=\left(55,12\right)$ yielded a KS distance of 0.31, compared to 0.58 with $\left(k,\bar{\tau }\right)=\left(52.5,13\right)$ and 0.95 with $\left(k,\bar{\tau }\right)=\left(45,17\right)$.

We then compared distributions of the ratio of modeled to empirical fluctuations in global network topology between the actual and the surrogate structural connectivity data (Fig 3B). The surrogate data were constructed by randomly rewiring actual edges with preserving the nodal unweighted and weighted degrees and the level of global synchrony. In the surrogate data, the ratio of modeled to empirical fluctuations was smaller than that in the actual data (see Fig 3B), where the mean of the ratio distribution was 0.52 (mean participation coefficient) and 0.49 (modularity). This result suggests that the network organization of structural connectivity contributed to the generation of fluctuations in global network topology of functional connectivity at magnitudes that resembled those observed empirically.

It should be mentioned that the magnitude of fluctuations in global network topology did not consistently decrease with a larger number of nodes. Details of our examination of the magnitude of fluctuations with a larger number of nodes are presented in S2 File and S2 Fig.

### Comparison of network states

#### Network metrics

**Fig 4:**
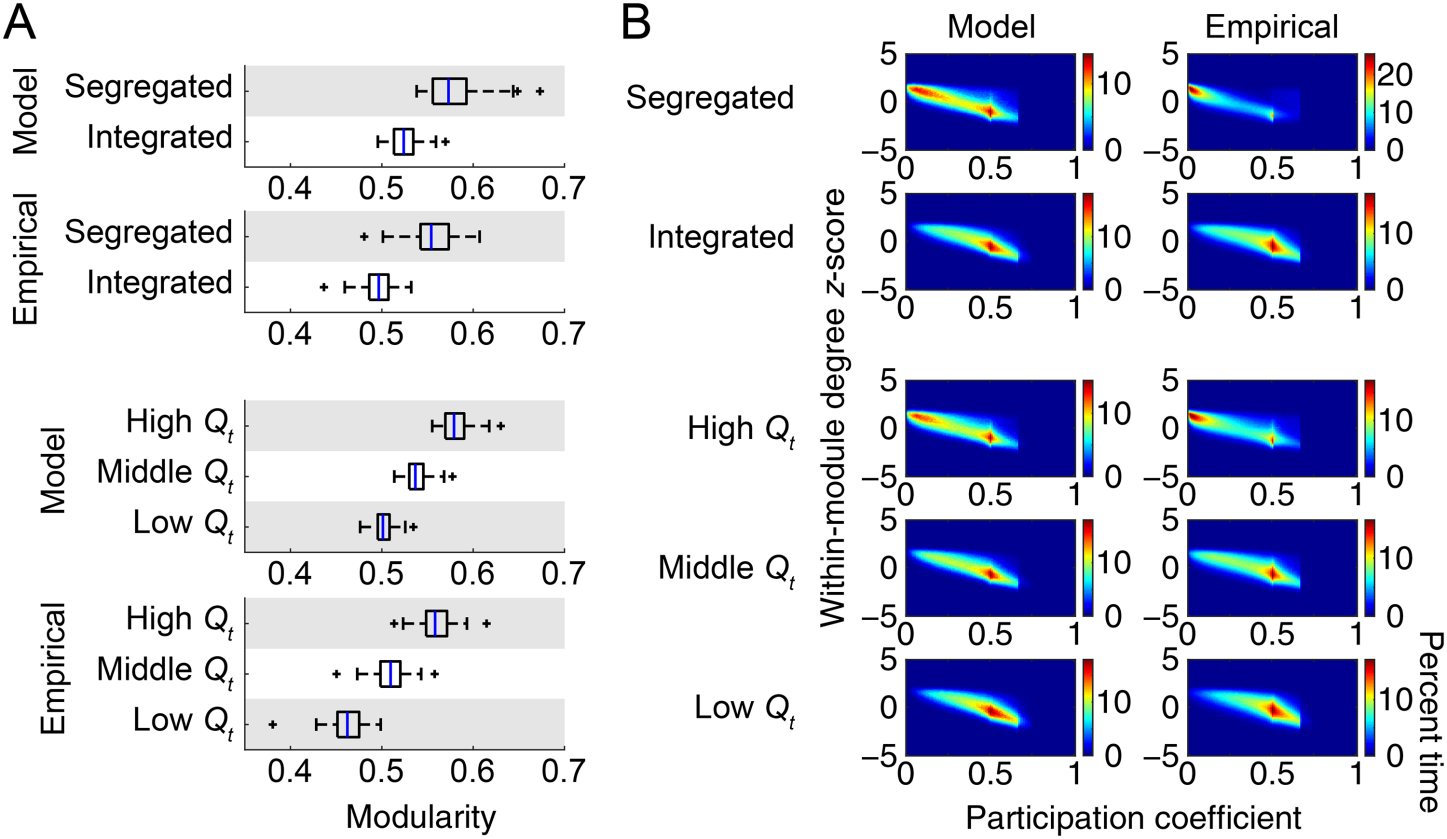
Network metrics averaged for each network state. (A) Modularity *Q*_*t*_ averaged for each network state within each individual sample (model) or subject (empirical). (B) Joint histograms of within-module degree *z*-score ***z***_*t*_ and participation coefficient ***P***_*t*_ over all nodes averaged for each network state. The percent time is derived from joint histograms of all samples (model) or subjects (empirical).

With the parameter set selected in the second stage of model parameter search, we compared network states of functional connectivity between the modeled and empirical data. We first compared network metrics used for determining network states, i.e., modularity *Q*_*t*_, within-module degree *z*-score ***z***_*t*_, and participation coefficient ***P***_*t*_. The metric *Q*_*t*_ averaged for each network state is shown in Fig 4A. Changing patterns in *Q*_*t*_ over network states were consistent in the modeled and empirical data. The magnitude of *Q*_*t*_ was also in a similar range in the modeled and empirical data—*Q*_*t*_ averaged over all instances was 0.54 in the modeled data and 0.51 in the empirical data. The mode and the median of the number of detected modules by modularity maximization were three in all network states, both in the modeled and empirical data.

In Fig 4B, we show joint histograms of ***z***_*t*_ and ***P***_*t*_ over all nodes averaged for each network state. The joint histograms had two peaks at around ***P***_*t*_ = 0 and 0.5 both in the modeled and empirical data. These two peaks were relatively less separable in the modeled data when clustering time windows into the segregated and integrated states. Nevertheless, overall spatial patterns in the joint histograms were common across the modeled and empirical data.

#### Temporal metrics

**Fig 5:**
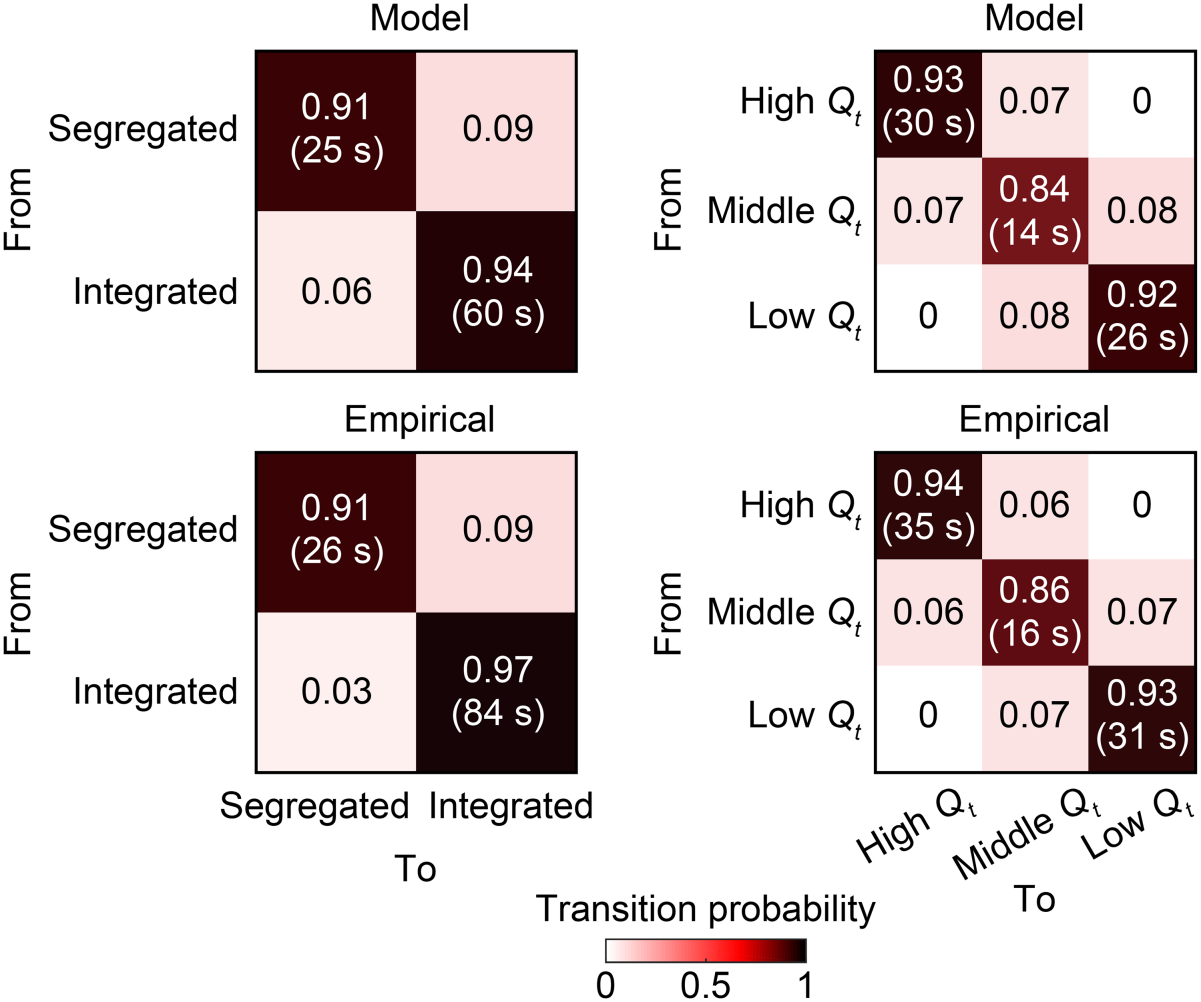
Temporal metrics of network states. The transition probability of each pair of network states is shown in the corresponding entry of matrices. The mean dwell time of each network state is presented in a parenthesis in the corresponding diagonal entry. Both temporal metrics are averaged over samples (model) or subjects (empirical).

We then compared temporal metrics that characterize the transition dynamics of network states. In Fig 5, we present transition probability of each pair of network states, together with mean dwell time of each network state. This figure demonstrates that empirical transition probability matrices and mean dwell time were highly reproducible in the modeled data.

#### Spatial patterns: Within/Between task-positive and task-negative networks

Next, we compared spatial patterns of functional connectivity during each network state between the modeled and empirical data, with a particular focus here on changes in functional connectivity within/between task-positive and task-negative networks.

**Fig 6:**
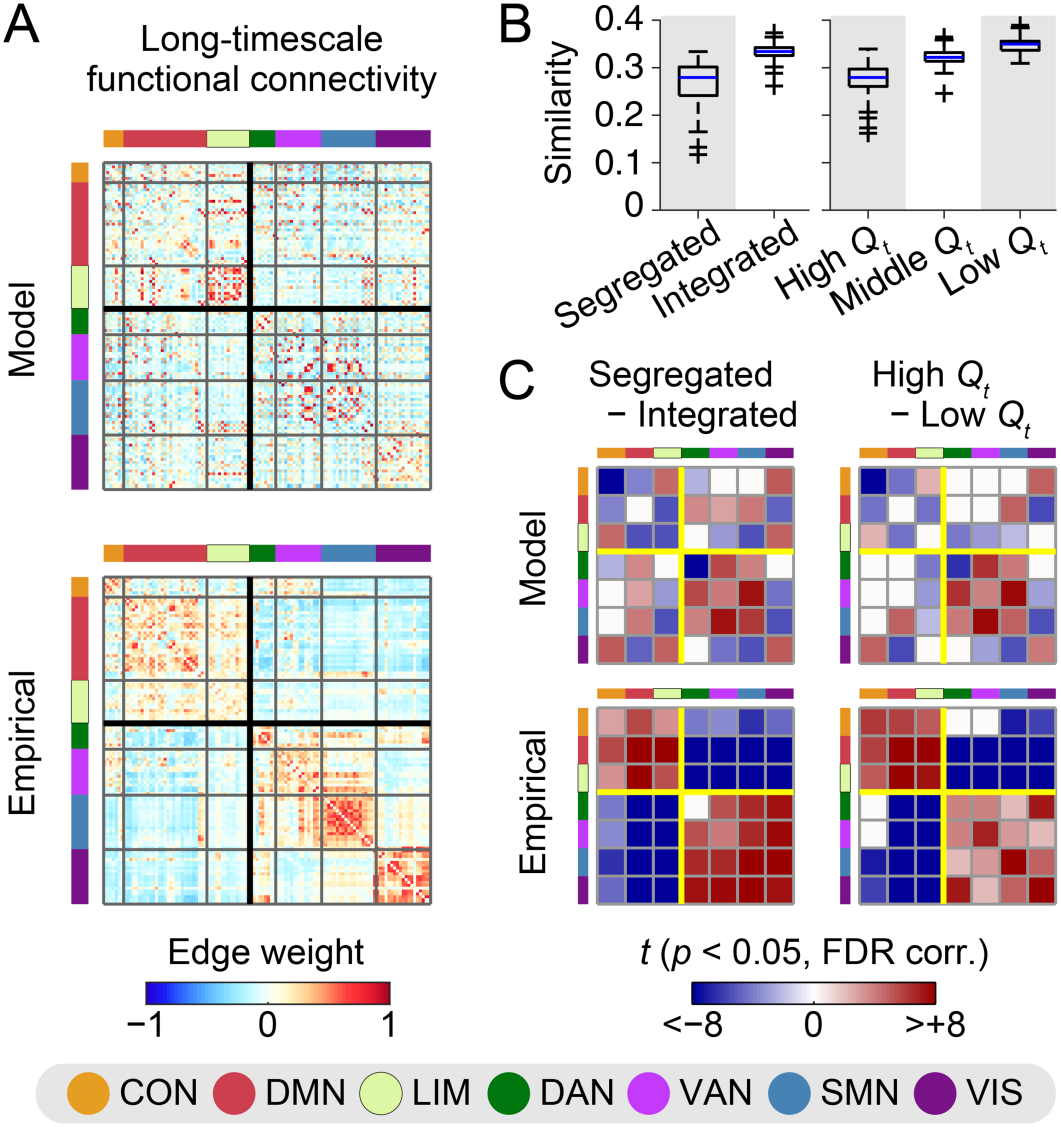
Spatial patterns of functional connectivity with respect to task-positive and task-negative networks. (A) Long-timescale functional connectivity of the modeled and empirical data (top: model; bottom: empirical). The modeled connectivity was averaged over 100 samples simulated with the parameter set $\left(k,\bar{\tau }\right)=\left(55,12\right)$. The empirical connectivity was averaged over 84 subjects. The black thick square lines in connectivity matrices indicate a partition dissociating task-negative (CON, DMN, LMN) and task-positive (DAN, VAN, SMN, VIS) networks. (B) The similarity of modeled and empirical centroids of time-resolved functional connectivity during each network state. The similarity was assessed between modeled centroids in each simulation sample and empirical centroids averaged over subjects. The similarity was quantified by the correlation coefficient of centroid’s functional connectivity weights over edges with direct structural connections. (C) Between-state differences in centroid’s functional connectivity weights (left: segregated state minus integrated state; right: high modularity period minus low modularity period; top: model; bottom: empirical). The between-state differences of weights were averaged within each pair of the seven network components in [50] and are presented in the matrices as *t*-scores if they were significant (*p* < 0.05, FDR corrected). The yellow square lines show the partition separating task-negative and task-positive networks.

In Fig 6A, we show long-timescale functional connectivity matrices averaged over samples (model) or subjects (empirical). In the empirical data, we confirmed canonical spatial patterns of functional connectivity, where positive (respectively, negative) edge weights were mainly found within (respectively, between) task-negative (CON, DMN, LIM) and task-positive (DAN, VAN, SMN, VIS) networks. These patterns can also been seen in the empirical connectivity matrix in S3A Fig, where nodes were sorted based on a partition that was obtained by applying modularity maximization to group-level empirical long-timescale functional connectivity (see S3B Fig for the partition). On the other hand, although moderate similarity scores were obtained in Fig 2A, the canonical spatial patterns observed in the empirical data were less evident in the modeled data. This discrepancy in the spatial patterns can be explained in part by the lack of reproducibility of the edge weight distributions within and between hemispheres (S4 Fig). Edge weights within and between hemispheres were similarly distributed in the empirical data, whereas positive (respectively, negative) edge weights were more frequently found within (respectively, between) hemispheres in the modeled data.

The centroid of time-resolved functional connectivity during each network state exhibited spatial connectivity patterns similar to those presented in Fig 6A when the centroids were averaged over samples (model) or subjects (empirical). While underlying spatial patterns were relatively common across network states, the contrast of spatial patterns was strengthened during the segregated state and the high modularity period. These network states were associated with greater global absolute functional connectivity both in the modeled and empirical data, compared to the integrated state and the low modularity period. The global absolute functional connectivity during the (segregated, integrated) state was (0.29 ± 0.07 [SD], 0.22 ± 0.01) in the modeled data (Cohen’s *d* = 1.4) and (0.26 ± 0.04, 0.19 ± 0.02) in the empirical data (*d* = 2.1), and that during the (high, low) modularity period was (0.27 ± 0.03, 0.22 ± 0.01) in the modeled data (*d* = 2.3) and (0.24 ± 0.03, 0.18 ± 0.02) in the empirical data (*d* = 2.2). Spatial patterns in the centroids have some variability over simulation samples, especially in the segregated state and the high modularity period. Modeled individual-level centroids of these two network states were less similar to empirical centroids averaged over subjects, compared to the integrated state and the low modularity period (Fig 6B). The variability of spatial patterns has also been reported in empirical individual-level centroids over subjects [33].

Then, to look into connectivity changes across network states in terms of task-positive and task-negative networks, we computed between-state differences in centroid’s functional connectivity weight averaged within each pair of the seven intrinsic network components [50]. We show *t*-scores of these between-state differences in Fig 6C to take into account the variability of centroids over samples and subjects. We confirmed that functional connectivity during the segregated state and the high modularity period of the empirical data increased within and decreased between task-negative and task-positive networks, exhibiting two major subdivisions as was reported in [12]. In contrast, this empirical finding was only partially reproducible from the modeled data. Examples of reproducible changes observed during the segregated state and the high modularity period were increased functional connectivity within the VAN + SMN, between the DAN and the VAN + SMN, and within the VIS, and decreased functional connectivity between the DMN and the VIS and between the LIM and the VAN. Non-reproducible major features were increased functional connectivity within the task-negative networks, within the DAN, and between the VIS and the other task-positive networks, and decreased functional connectivity between the VIS and the CON + LIM and between the DMN and the task-positive networks other than the VIS. When the between-state differences were evaluated without taking into account interhemispheric connections, some of these features (increased functional connectivity within the DMN and between the CON and all the task-negative networks including itself) were also observed in the modeled data (see S5 Fig). However, the model did not reproduce the decoupling between task-positive and task-negative systems during the segregated state and the high modularity period. The between-state differences averaged based on the partition derived from modularity maximization were presented in S3C Fig. This figure also shows that increased/decreased dissociation of task-positive and task-negative networks was not reproduced in the modeled data.

#### Spatial patterns: The similarity to structural connectivity

Lastly, we focused on the similarity of functional connectivity to structural connectivity and compared changes in this relationship across network states between the modeled and empirical data.

**Fig 7:**
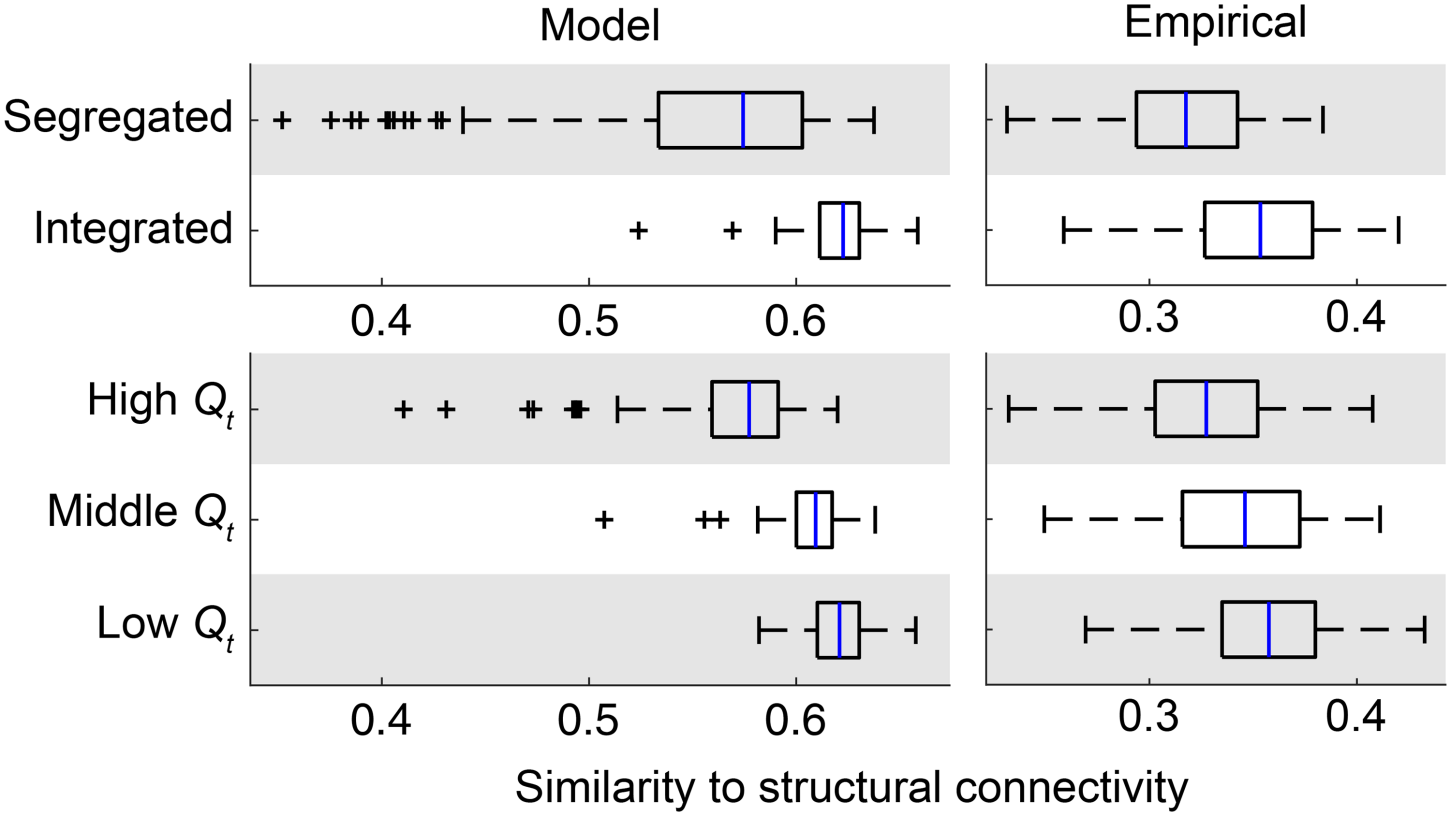
Spatial patterns of functional connectivity with respect to its similarity to structural connectivity. The similarity between group-level structural connectivity and the centroid of time-resolved functional connectivity during each network state in each sample (model) or subject (empirical) was presented for the segregated and integrated states (top) and the high, middle, and low modularity periods (bottom). The similarity for modeled functional connectivity is shown in the left side and the similarity for empirical functional connectivity is shown in the right side.

Fig 7 shows the similarity between structural connectivity and the centroid of time-resolved functional connectivity during each network state. Compared to the segregated state and the high modularity period, functional connectivity during the integrated state and the low modularity period exhibited greater similarity to structural connectivity in the empirical data (Fig 7, right; *d* = 1.0 both for segregated/integrated and high/low modularity comparisons), as was previously shown in [33]. While the magnitude of the similarity to structural connectivity in the modeled data was overall greater than that observed in the empirical data, the changes in the similarity to structural connectivity across network states was reproducible from the modeled data (Fig 7, left). As observed in the empirical data, greater similarity to structural connectivity was associated with the integrated state (*d* = 1.3, compared to the segregated state) and with the low modularity period (*d* = 1.8, compared to the high modularity period) of modeled functional connectivity.

### Results with noise in simulations

The modeled data generated with noise in simulations yield main results similar to those obtained without noise. We confirmed that similar results were observed in the ratio of modeled to empirical fluctuations in global network topology (Fig 3B and S6A Fig), network metrics used for determining network states (Fig 4 and S6B and S6C Fig), temporal metrics of network states (Fig 5 and S6D Fig), and spatial patterns of functional connectivity and their changes across network states (Figs 6, 7 and S7 Fig).

## Discussion

In this study, we evaluated fluctuations in global network topology of modeled functional connectivity by comparing them with fluctuations in the empirical data from multiple perspectives. For modeling of regional cortical activity, we used a network of phase oscillator models coupled based on brain structural connectivity. After confirming moderate similarity of long-timescale and time-resolved functional connectivity between the modeled and empirical data, we fitted two free parameters of this network model, namely the global coupling constant and the conduction velocity (or the mean time delay), so as to maximize the reproducibility of empirical fluctuations in metrics quantifying network integration and modularity of functional connectivity. With the parameter set selected, we showed that fluctuations in both mean participation coefficient and modularity of modeled functional connectivity have magnitudes of more than 80% of those observed in the empirical counterparts and that the stationary model also reproduced empirical modularity and joint histogram of within-module degree *z*-score and participation coefficient during each network state, as well as empirical transition probability and mean dwell time of network states. We then compared spatial patterns of functional connectivity and their changes across network states, especially those reported in [12, 33], between the modeled and empirical data. We demonstrated that the between-state changes in functional connectivity with respect to the relationship of task-positive and task-negative networks were only partially reproducible, while the between-state changes in the similarity to structural connectivity were more fully reproducible in our model setting.

By directly examining the reproducibility of fluctuations in global network topology, the present study contributes to advance our understanding about the degree to which a stationary model simulating resting-state activity can explain spatiotemporal patterns of empirical fluctuations between segregated and integrated topology [10] or high and low modularity topology [12]. Since such empirical fluctuations have received increasing attention because of recent reports on their relationships to human cognition and behavior [10, 13, 14], there is presently a strong need for modeling studies to explore their generative mechanisms by quantitatively examining the predictability of computational models to the empirically observed topological fluctuations. However, no direct comparison of modeled and empirical data has been made in existing works that have studied modeled fluctuations in global network metrics of segregation and integration [8, 29]. In those studies, it is difficult to see how well the generated modeled fluctuations in global network topology reflect the real empirical fluctuations. In many other studies, comparison of modeled and empirical data has been performed in investigating modeled long-timescale and time-resolved functional connectivity [9, 19–27], whereas these investigations have not been extended so far to the fluctuations in global network metrics of segregation and integration. In this study, we extended these two lines of existing studies by quantitatively comparing modeled and empirical fluctuations in these global network metrics. Our comparison of the fluctuations revealed comprehensively the extent to which a stationary dynamic model of spontaneous brain activity can reproduce empirical features of fluctuations in segregation and integration, and thereby provides new insights into generative mechanisms of topological fluctuations that have been recently associated with human cognition and behavior.

In our comparison of fluctuations in global network topology between the modeled and empirical data, we found both reproducible and non-reproducible features. Reproducible features include the overall magnitude of fluctuations in mean participation coefficient [10] and in modularity [11, 12], the quantity of metrics quantifying network integration and modularity during each network state, temporal metrics characterizing state transition dynamics, between-state changes in the relationship of functional connectivity to structural connectivity [33], and a limited part of the between-state changes in functional connectivity within/between task-positive and task-negative networks [12]. The fact that a number of properties about the network-level fluctuations were shown to be reproducible suggests that, to some extent, the modeled fluctuations in global network topology may reflect actual network-level dynamics in the resting brain. On the other hand, we found that between-state changes in functional connectivity occurring within/between task-positive and task-negative networks were overall not well captured in the modeled data, even when the changes across network states were evaluated by only taking into account the intra-hemispheric connections. Potential limiting factors include the lack of an explicit physiological model of context-dependent and transient dynamics, and/or possible errors in estimation of brain structural connectivity [63]. These factors may also diminish the relationship between modeled and empirical long-timescale functional connectivity in this study as well as in previous studies [19–23]. These two issues will be further discussed later.

The finding that the magnitudes of fluctuations in mean participation coefficient and modularity of the empirical data were preserved in our modeled data simulated with a time-invariant dynamical system suggests that explicit modeling of non-stationary dynamics is not necessarily required for an appearance of the fluctuations themselves. In very recent studies by Glomb et al. [26, 27], another stationary dynamic model has also largely reproduced slow alternations between more and less activated states of functional connectivity components extracted by tensor decomposition. Interestingly, randomly rewired structural connectivity patterns yielded greatly reduced fluctuations in this study, indicating that network patterns of anatomical connections contribute in part to the emergence of such fluctuations. These findings echo earlier observations concerning the emergence of additional (longer) time scales in modeled functional connectivity, and a role of anatomical connections in shaping fluctuations in functional couplings [18].

In contrast, the non-reproducible features that we observed in changes in functional connectivity across network states suggest that additional factors, such as physiological mechanisms that drive non-stationary dynamics and/or greater accuracy in mapping structural connections, are needed to more fully replicate the observed fluctuations in network integration and modularity. In addition to the non-reproducible features, we also observed that the magnitude of fluctuations in global network topology of the modeled data was slightly (on average 10–20%) smaller than the magnitude in the empirical data (Fig 3B). Moreover, it has also been reported that the stationary dynamic model in [27] cannot fully reproduce the dynamics of modulations in time-resolved functional connectivity. Modeling of the non-stationarity may help in further improving the matching between the modeled and empirical data in reproducing such fluctuations and modulations. In regard to limitations inherent in mapping structural connections (see also **Methodological considerations**), replacing structural connectivity with directed effective connectivity [76] may solve the problem of fully reproducing the empirical spatial patterns. This approach has been taken by the recent studies [26, 27], improving the reproducibility of spatial patterns associated with empirical fluctuations.

A promising approach for the purpose of incorporating the non-stationarity is to model the modulation of neural gain mediated by neuromodulatory brain systems [77]. Shine and colleague have shown that fluctuations in mean participation coefficient are correlated with fluctuations in pupil diameter, a surrogate measure for the activity of neuromodulatory systems [78], in an empirical study [10] and that manipulating modeled neural gain gives rise to abrupt changes in mean participation coefficient in a modeling study [29]. What has not been achieved so far in this line of research is directly modeling the dynamics of neural gain control and integrating this model component into the current modeling framework for the simulation of resting-state cortical activity. Comparison of the modeled and empirical data with an explicit modeling of gain control dynamics may provide a better understanding about the generative mechanism of empirical fluctuations in network integration and modularity.

### Methodological considerations

The present study has several methodological limitations. First, while white matter tractography using DWI data is a primary technique for estimating human structural connectivity, it can be prone to inaccuracies [79, 80] especially for estimating connections between hemispheres [63]. In our study, errors in structural connectivity may have influenced the simulations of resting-state cortical activity, as is suggested by our observation that the magnitude of fluctuations was not accurately reproduced in the modeled data simulated with surrogate structural connectivity. Furthermore, taking into account only intra-hemispheric functional connections for evaluating patterns in changes between network states exhibited within-system coherence, but failed to show between-system decoupling. Errors in estimating structural connections (and thus in modeling functional connections) between hemispheres may have contributed to this negative result. In spite of these limitations, we believe that the emergence of fluctuations in global network topology of modeled functional connectivity were not a consequence of inaccuracies inherent in tractography, since the fluctuations themselves have also been observed in modeled data simulated with tract-tracing-based macaque structural connectivity retrieved from the CoCoMac database [8, 29]. Nevertheless, future research is needed to compare the modeled and empirical fluctuations without potential biases due to tractography, using e.g. the CoCoMac structural connectivity data and empirical macaque rs-fMRI data.

Second, assessing the non-stationarity of coactivation patterns with rs-fMRI has its own limitations. Existing studies have shown that the non-stationarity of (pairwise) functional connectivity is difficult to be detected from an insufficient amount of rs-fMRI data [81] and is also subject to potential confounds such as head movements and/or physiological noise [82]. Especially the latter issue is needed to be carefully addressed when quantifying the magnitude of fluctuations in global network topology of empirical functional connectivity. We applied extensive artifact reduction methods to the empirical rs-fMRI data employed in this study. With this dataset, we previously demonstrated that no consistent relation was found in fluctuations in mean participation coefficient to either head motion or respiration [33]. Therefore, effects of artifacts on our evaluation of the magnitude of empirical fluctuations should be limited.

Third, we modeled the dynamics of regional cortical activity using rather simple Kuramoto oscillators, in which the collective behavior of neural populations in each region was described by a single phase variable. The reason for choosing the Kuramoto model is computational efficiency to enable a systematic model parameter search with sufficient numbers of simulation samples per each parameter set, especially for the analysis of fluctuations in global network topology. Current computational resources do not allow replacing this model with more complex models, such as a neural mass model employed in [8, 18, 19], in which local neural dynamics are described by multiple nonlinear differential equations. In support of our model choice, previous studies have demonstrated that the prediction accuracy of long-timescale functional connectivity of the Kuramoto model is comparable to that of other major computational models including the above-mentioned neural mass model [23, 63].

Fourth, we used group-level structural connectivity for generating modeled resting-state cortical activity, although it would be ideal to use individual structural connectivity instead to conduct comparison of the modeled and empirical data within each individual. Resting-state activity has recently been simulated using individual structural connectivity in e.g. [83] for the purpose of exploring individual differences in cognition. Nevertheless, conducting all the analyses in this study using individual structural connectivity is infeasible due to excessive demands on computation time, especially for searching model parameters of every single subject. Performing the analyses in this study using individual structural connectivity for tens of subjects may become feasible as computing power increases.

### Future directions

Future work is needed to gain further insights into the generative mechanism of fluctuations in global network topology of functional connectivity. An interesting direction of future research is to try to uncover local features of transient activity propagations underlying these global network-level fluctuations. Revealing such local features may allow us to understand how the global fluctuations in network integration and modularity emerge from sequences of time-resolved regional activities over the entire brain. Propagations of regional activity can be investigated using point-process analysis [84, 85]. Another important future direction is to provide a mechanistic understanding of the global network-level fluctuations by manipulating elements in the network model, for example, introducing lesions in structural connectivity that wires local dynamic models [62, 86]. Examining the effects of manipulations on the outcomes would contribute to elucidate model elements necessary for the emergence of fluctuations in network integration and modularity. We will pursue these avenues of research in future work, with continued emphasis on the generative aspects of fluctuations in global network topology.

## Supporting information

S1 Fig Cortical parcellation produced by a subdivision of the Desikan-Killiany atlas. The numbers placed on cortical parcels indicate the order of nodes in connectivity matrices in main figures. The colors behind these numbers present the maximally-overlapped network component of the seven network parcellation in [50].
(TIF)

S2 Fig Distributions of the magnitude of fluctuations in global network metrics with a larger number of nodes. The distributions obtained from 100 simulations with the number of nodes *N* = 114 are shown in light red and the distributions from *N* = 219 are shown in dark red (the overlapped areas are shown in red). Distributions for mean participation coefficient are presented in the left panel and distributions for modularity are in the right panel.
(TIF)

S3 Fig Spatial patterns of functional connectivity with a partition derived from empirical long-timescale functional connectivity. (A) Long-timescale functional connectivity of the modeled and empirical data (cf. Fig 6A), in which nodes are sorted based on the partition shown in (B). This partition was obtained by applying modularity maximization to group-level empirical long-timescale functional connectivity. The communities of nodes colored by blue, light blue, and cyan overlap areas of the DMN, SMN, and VIS, respectively, in [50]. (C) Between-state differences in centroid’s functional connectivity weights (cf. Fig 6C). The between-state differences of weights were averaged within each pair of modules in the partition shown in (B).
(TIF)

S4 Fig Edge weight distributions of long-timescale functional connectivity. Distributions of edge weights within hemispheres are shown in red and those between hemispheres are shown in blue (top: model; bottom: empirical).
(TIF)

S5 Fig Between-state differences in centroid’s functional connectivity weights without taking into account interhemispheric connections. The differences between the segregated and integrated states are shown on the left and those between the high and low modularity periods are shown on the right (top: model; bottom: empirical) (cf. Fig 6C).
(TIF)

S6 Fig Results with noise in simulations: The magnitude of fluctuations, network metrics, and temporal metrics. (A) The ratio of modeled to empirical SD of mean participation coefficient (left) and modularity (right) (cf. Fig 3B). (B) Modularity during each network state (cf. Fig 4A). (C) Joint histograms of within-module degree *z*-score and participation coefficient during each network state (cf. Fig 4B). (D) Transition probability and mean dwell time of network states (cf. Fig 5).
(TIF)

S7 Fig Results with noise in simulations: Spatial patterns of functional connectivity and their between-state changes. (A) Long-timescale functional connectivity (cf. Fig 6A). (B) The similarity of modeled and empirical centroids of time-resolved functional connectivity during each network state (cf. Fig 6B). (C) Between-state differences in centroid’s functional connectivity weights (cf. Fig 6C). (D) The similarity between structural connectivity and the centroid of time-resolved functional connectivity during each network state (cf. Fig 7).
(TIF)

S1 File Numerical datasets that underlie all figures in the main text. (XLSX)

S2 File Examination of the magnitude of fluctuations with a larger number of nodes. (PDF)
