# Supplementary figures and images for: Comparison of fluctuations in global network topology of modeled and empirical brain functional connectivity
Source: PLoS Comput Biol. 2018 Sep 25;14(9):e1006497. doi: 10.1371/journal.pcbi.1006497 (PMC6173440; doi:10.1371/journal.pcbi.1006497)

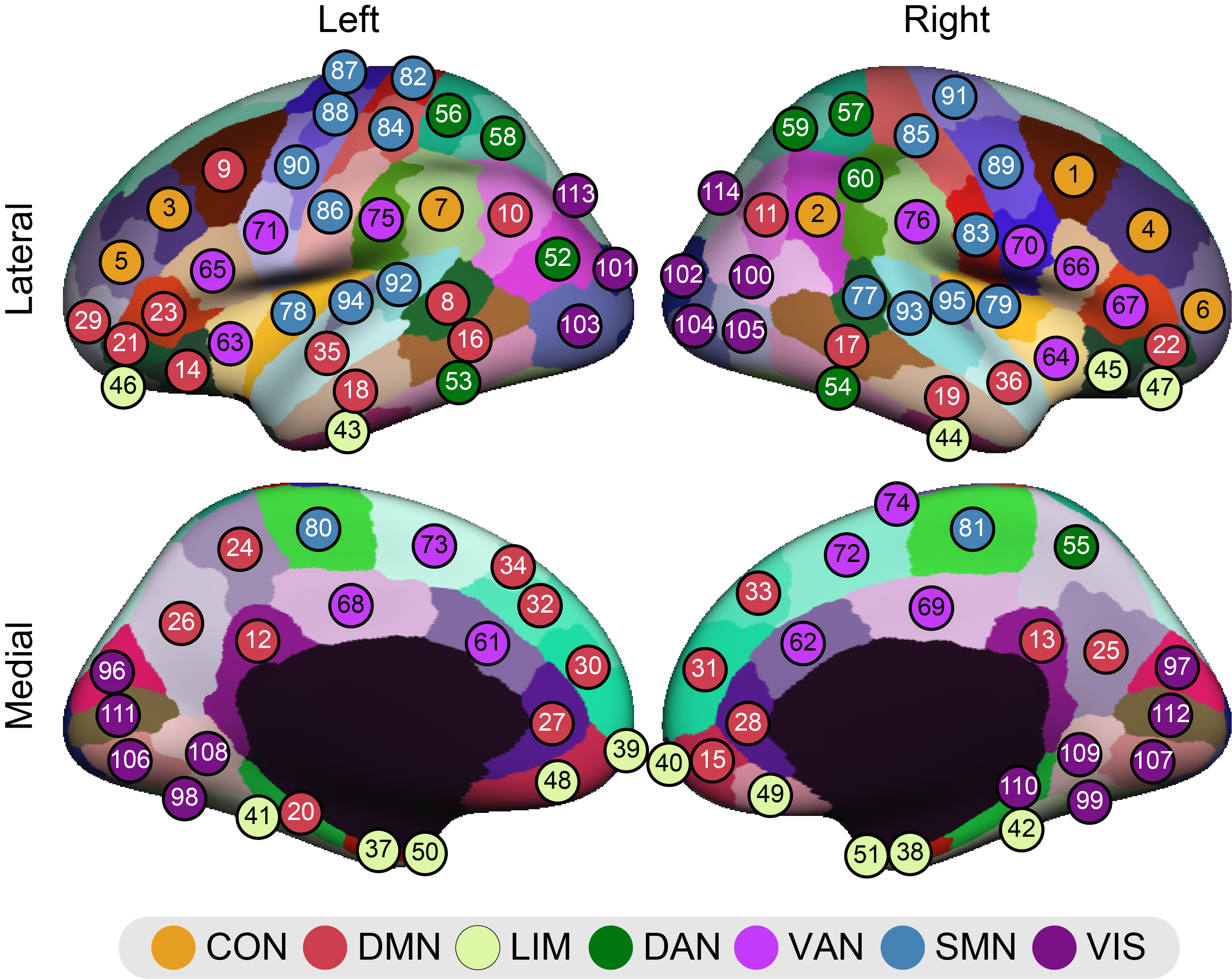

Supplement: S1 Fig — The numbers placed on cortical parcels indicate the order of nodes in connectivity matrices in main figures. The colors behind these numbers present the maximally-overlapped network component of the seven network parcellation in [50]. (TIF) [file pcbi.1006497.s001.tif]

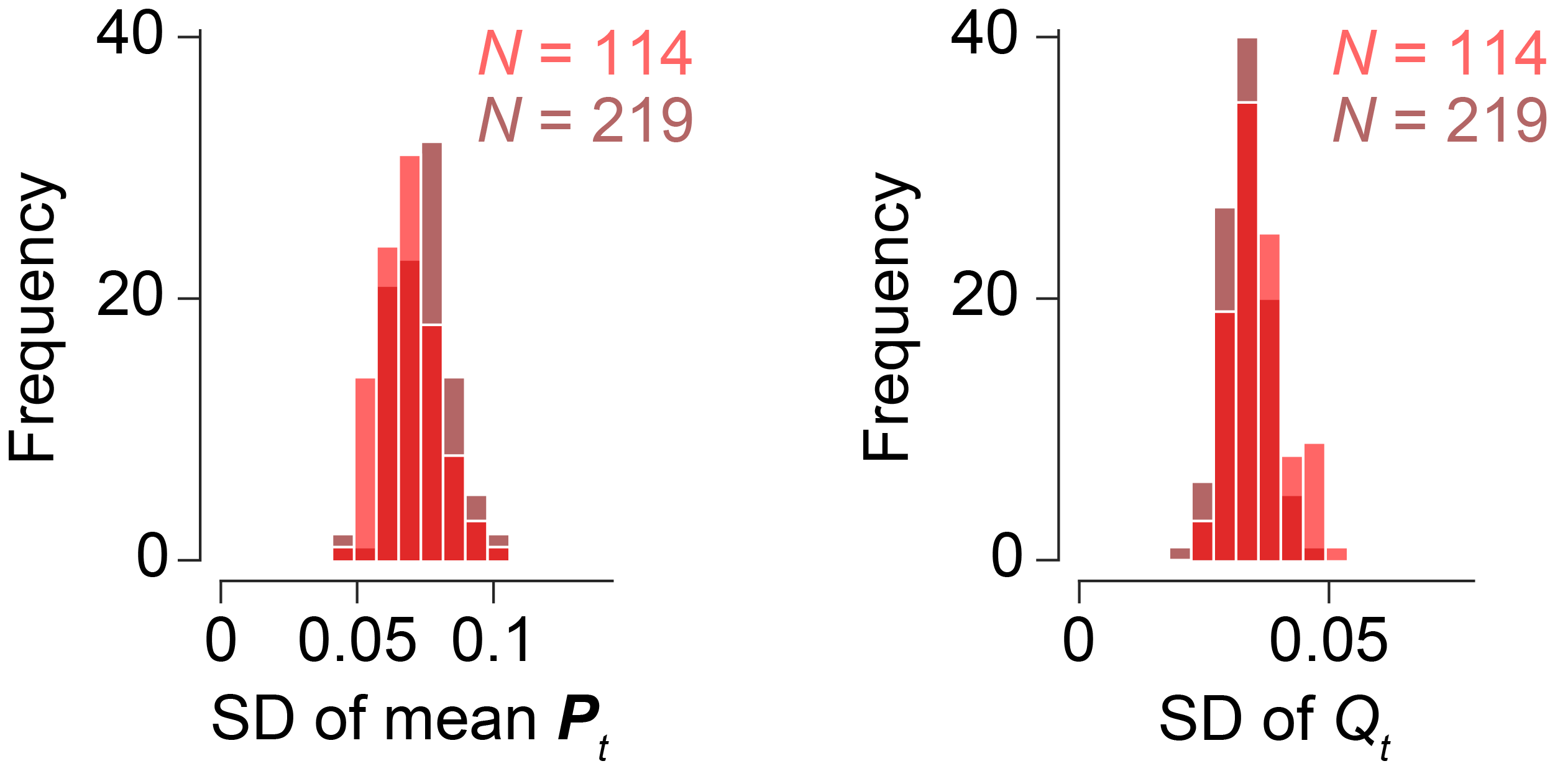

Supplement: S2 Fig — The distributions obtained from 100 simulations with the number of nodes N = 114 are shown in light red and the distributions from N = 219 are shown in dark red (the overlapped areas are shown in red). Distributions for mean participation coefficient are presented in the left panel and distributions for modularity are in the right panel. (TIF) [file pcbi.1006497.s002.tif]

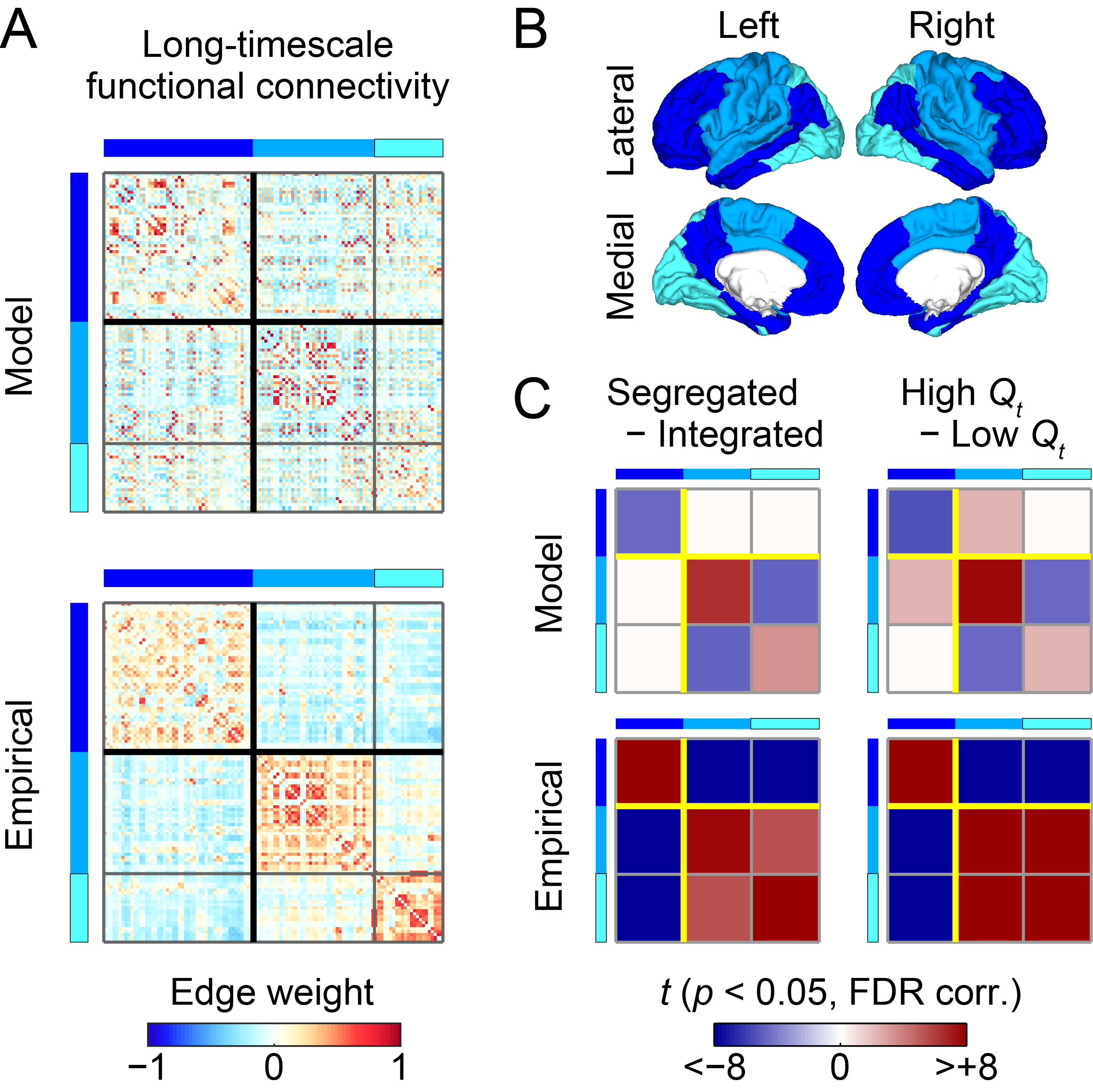

Supplement: S3 Fig — (A) Long-timescale functional connectivity of the modeled and empirical data (cf. Fig 6A), in which nodes are sorted based on the partition shown in (B). This partition was obtained by applying modularity maximization to group-level empirical long-timescale functional connectivity. The communities of nodes colored by blue, light blue, and cyan overlap areas of the DMN, SMN, and VIS, respectively, in [50]. (C) Between-state differences in centroid’s functional connectivity weights (cf. Fig 6C). The between-state differences of weights were averaged within each pair of modules in the partition shown in (B). (TIF) [file pcbi.1006497.s003.tif]

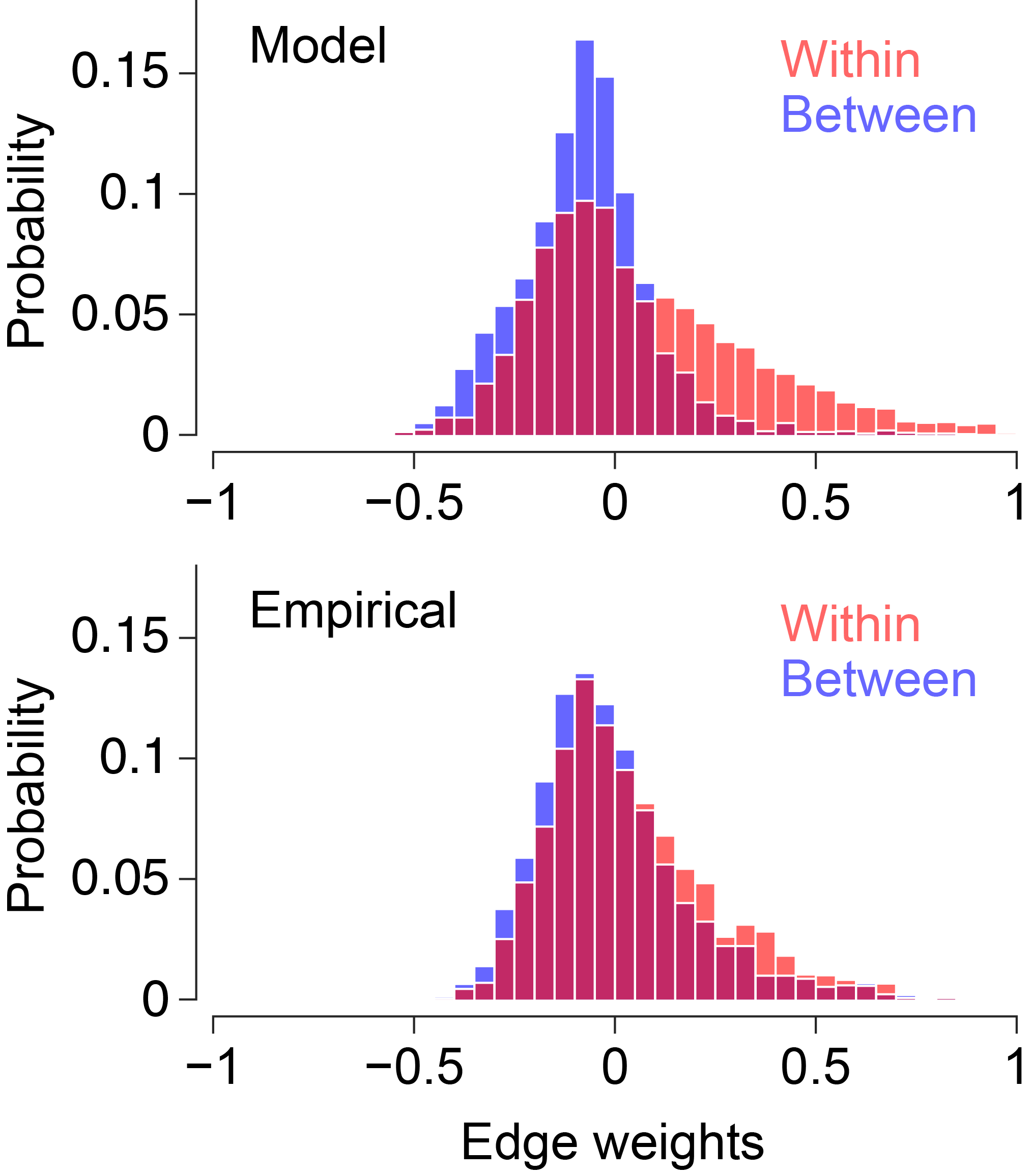

Supplement: S4 Fig — Distributions of edge weights within hemispheres are shown in red and those between hemispheres are shown in blue (top: model; bottom: empirical). (TIF) [file pcbi.1006497.s004.tif]

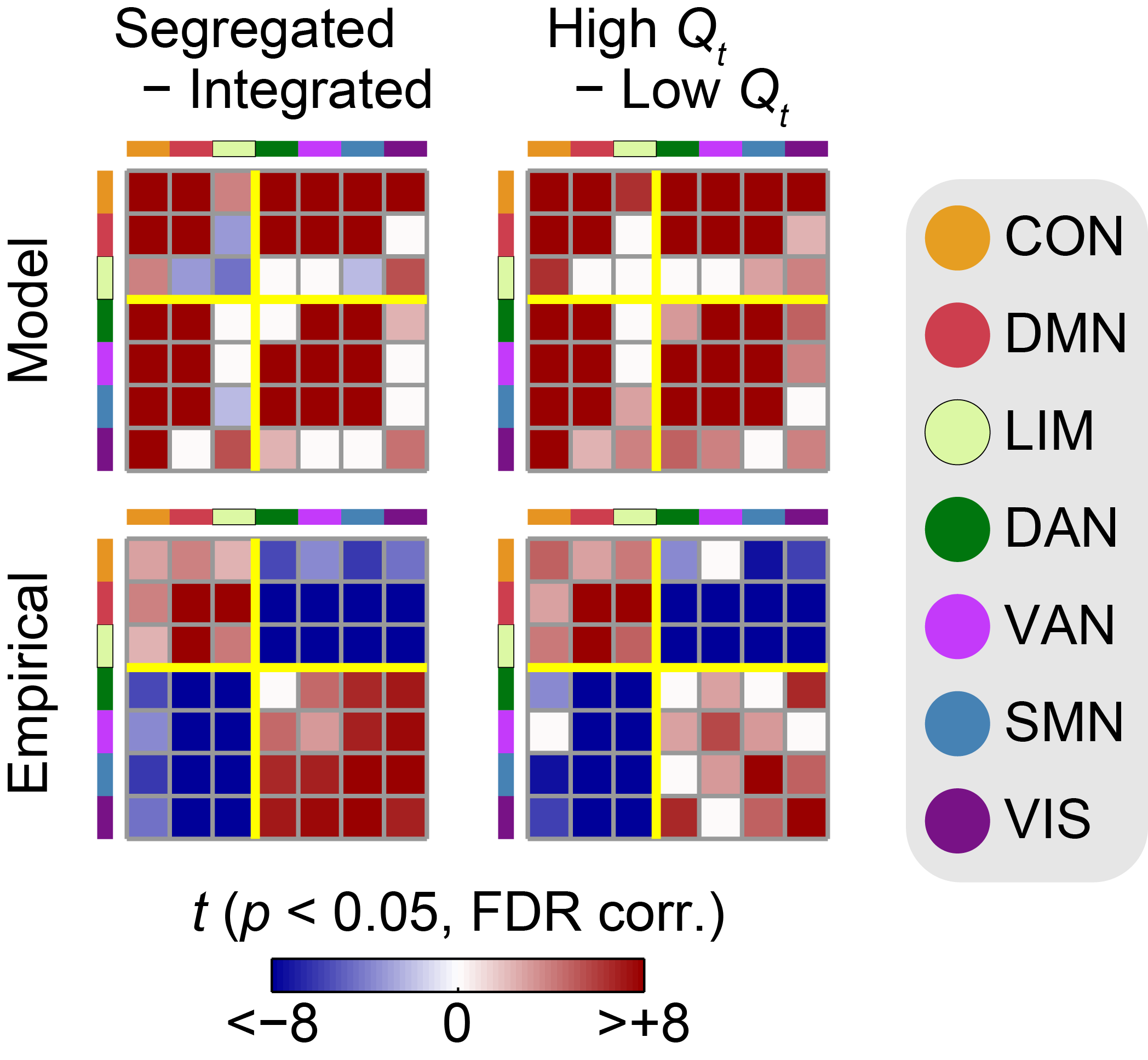

Supplement: S5 Fig — The differences between the segregated and integrated states are shown on the left and those between the high and low modularity periods are shown on the right (top: model; bottom: empirical) (cf. Fig 6C). (TIF) [file pcbi.1006497.s005.tif]

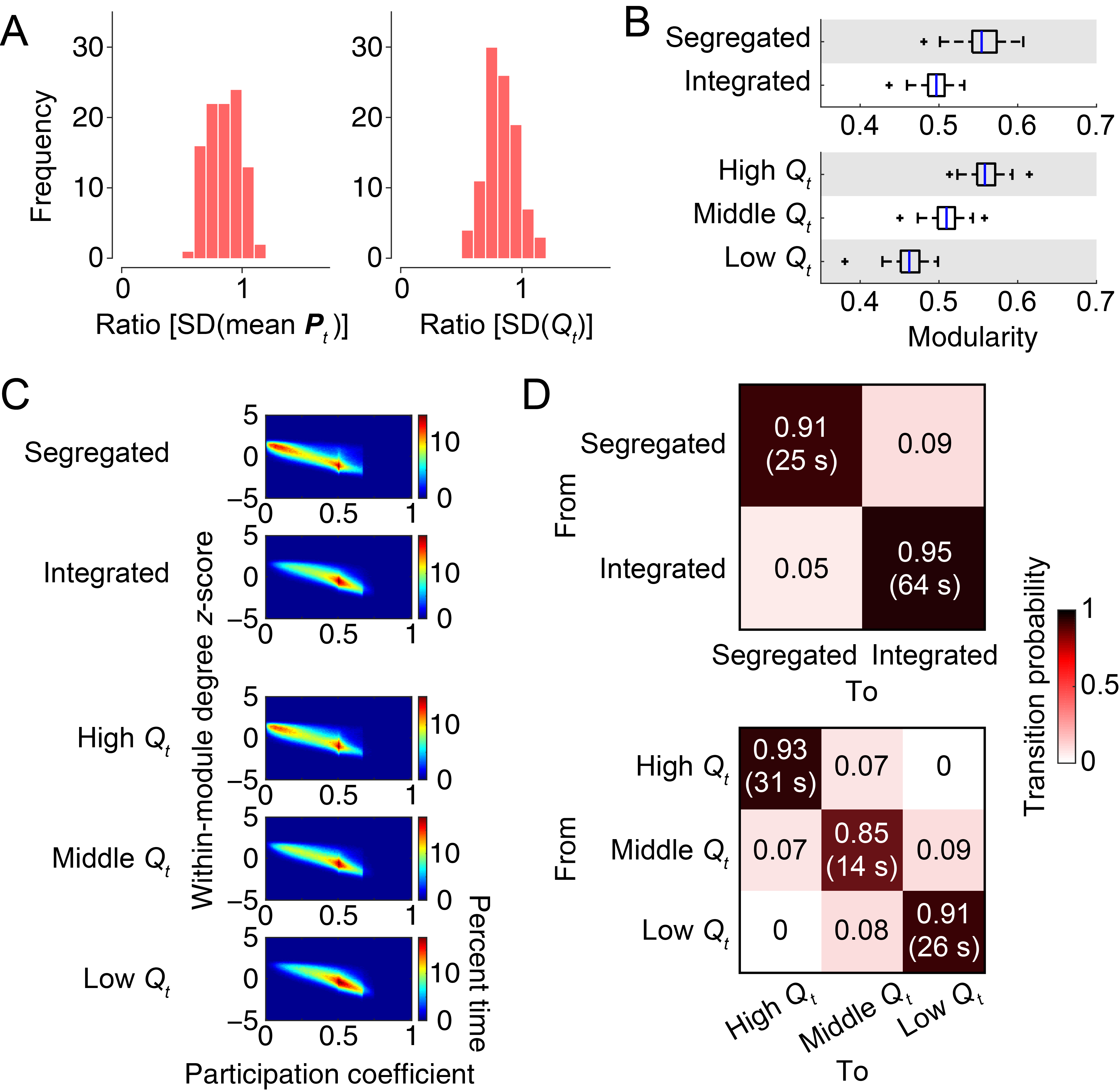

Supplement: S6 Fig — (A) The ratio of modeled to empirical SD of mean participation coefficient (left) and modularity (right) (cf. Fig 3B). (B) Modularity during each network state (cf. Fig 4A). (C) Joint histograms of within-module degree z-score and participation coefficient during each network state (cf. Fig 4B). (D) Transition probability and mean dwell time of network states (cf. Fig 5). (TIF) [file pcbi.1006497.s006.tif]

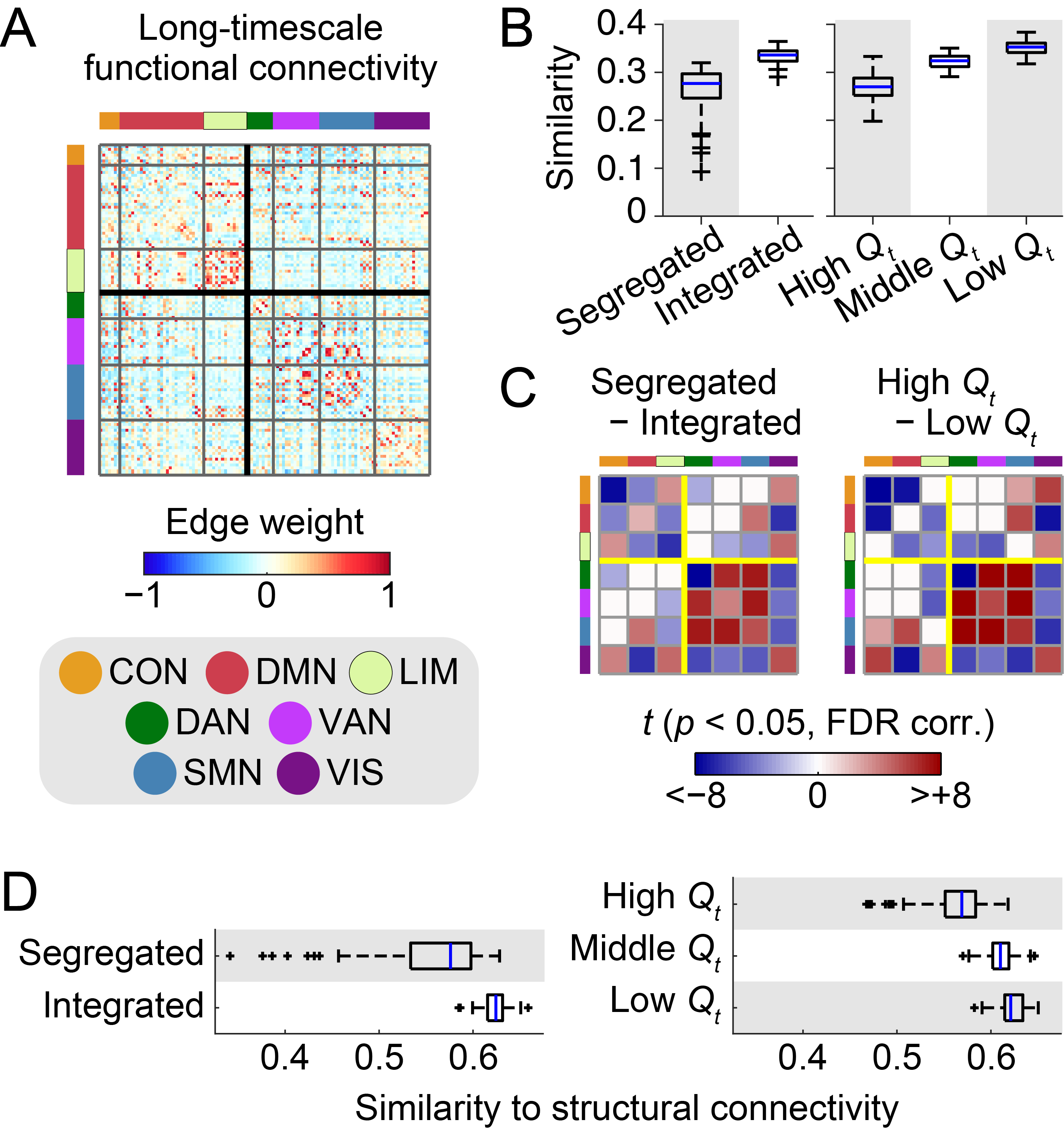

Supplement: S7 Fig — (A) Long-timescale functional connectivity (cf. Fig 6A). (B) The similarity of modeled and empirical centroids of time-resolved functional connectivity during each network state (cf. Fig 6B). (C) Between-state differences in centroid’s functional connectivity weights (cf. Fig 6C). (D) The similarity between structural connectivity and the centroid of time-resolved functional connectivity during each network state (cf. Fig 7). (TIF) [file pcbi.1006497.s007.tif]
