# Supplementary material for: Comparison of fluctuations in global network topology of modeled and empirical brain functional connectivity
Source: PLoS Comput Biol. 2018 Sep 25;14(9):e1006497. doi: 10.1371/journal.pcbi.1006497 (PMC6173440; doi:10.1371/journal.pcbi.1006497)
Supplement: S2 File — (PDF) [file pcbi.1006497.s009.pdf]

S2 File. Examination of the magnitude of fluctuations with a larger number of nodes

We examined whether or not the magnitude of fluctuations in global network topology of modeled functional connectivity decreases when the number of nodes N is set to a number greater than 114. To answer this question, we performed simulations using a group-level structural connectivity matrix with finer spatial resolution of the Desikan-Killiany atlas.

Methods

The structural connectivity matrix with a larger number of nodes was constructed using a parcellation derived from the atlas files *myatlas_125_lh.gcs* and *myatlas_125_rh.gcs* in the Connectome Mapper package. The number of nodes in this connectivity matrix was 219. Connectivity strength and fiber length were computed in the same manner as described in **Structural connectivity** in **Materials and methods**. We fitted the level of global synchrony between the two cases of the number of nodes 114 and 219 by varying the global coupling constant k, while we fixed the mean time delay $\bar{\tau}$ to the value used in $N = 114$ to reduce the computation time. The parameter k was varied over the range of 2.5–70 in steps of 2.5. For each value of k, we generated 10 simulation samples to compare the level of global synchrony (i.e., the mean of the order parameter) between the cases $N = 114$ and 219. After selecting k from which the closest level of global synchrony was observed, we newly generated 100 simulation samples with the selected k and compared the magnitude of fluctuations in mean participation coefficient and modularity between the two cases of the number of nodes.

Results

We first confirmed that the mean degree of structural connectivity matrix was preserved in the two cases (22 when $N = 114$; 23 when $N = 219$) and so was the level of global synchrony of simulated activity (the mean of the order parameter: 0.37 ± 0.02 when $N = 114$ and $k = 55$; $0.37 \pm 4.9 \times 10^{-4}$ when $N = 219$ and $k = 65$). Distributions of the magnitudes of fluctuations in global network metrics are shown in S2 Fig. This figure demonstrates that the magnitude was in a similar range in both cases of the number of nodes and did not consistently decrease when N was increased from 114 to 219. While we did not confirm this property with even larger number of nodes due to very high computational time, however, this result indicates that our conclusion regarding the magnitude of global fluctuations does not change when the number of nodes is set to a range typically used for simulating large-scale brain activity in the literature.
